# Optimization of surgical intervention outside the epileptogenic zone in the Virtual Epileptic Patient (VEP)

**DOI:** 10.1371/journal.pcbi.1007051

**Published:** 2019-06-26

**Authors:** Sora An, Fabrice Bartolomei, Maxime Guye, Viktor Jirsa

**Affiliations:** 1 Aix Marseille Univ, INSERM, INS, Inst Neurosci Syst, Marseille, France; 2 Aix Marseille Univ, CNRS, CRMBM UMR 7339, Marseille, France; Ghent University, BELGIUM

## Abstract

Studies to improve the efficacy of epilepsy surgery have focused on better refining the localization of the epileptogenic zone (EZ) with the aim of effectively resecting it. However, in a considerable number of patients, EZs are distributed across multiple brain regions and may involve eloquent areas that cannot be removed due to the risk of neurological complications. There is a clear need for developing alternative approaches to induce seizure relief, but minimal impact on normal brain functions. Here, we develop a personalized in-silico network approach, that suggests effective and safe surgical interventions for each patient. Based on the clinically identified EZ, we employ modularity analysis to identify target brain regions and fiber tracts involved in seizure propagation. We then construct and simulate a patient-specific brain network model comprising phenomenological neural mass models at the nodes, and patient-specific structural brain connectivity using the neuroinformatics platform The Virtual Brain (TVB), in order to evaluate effectiveness and safety of the target zones (TZs). In particular, we assess safety via electrical stimulation for pre- and post-surgical condition to quantify the impact on the signal transmission properties of the network. We demonstrate the existence of a large repertoire of efficient surgical interventions resulting in reduction of degree of seizure spread, but only a small subset of them proves safe. The identification of novel surgical interventions through modularity analysis and brain network simulations may provide exciting solutions to the treatment of inoperable epilepsies.

## Author summary

We propose a personalized in-silico surgical approach able to suggest effective and safe surgical options for each epilepsy patient. In particular, we focus on deriving effective alternative methods for those cases where EZs are inoperable because of issues related with neurological complications. Based on modularity analysis using structural brain connectivity from each patient, TZs that would be considered as surgical sites are obtained. The acquired TZs are evaluated by personalized brain network simulations in terms of effectiveness and safety. Through the feedback approach combining modularity analysis and brain network simulations, the optimized TZ options that minimize seizure propagation while not affecting normal brain functions are obtained. Our study has a great importance in that it demonstrates the possibility of computational neuroscience field being able to construct a paradigm for personalized medicine by deriving innovative surgical options suitable for each patient and predicting the surgical outcomes.

## Introduction

Epilepsy is a chronic neurological disorder that is defined by the occurrence of repetitive unexpected seizures. The epileptic seizures, characterized as abnormal synchronization of neural activities, originate in a specific brain region and propagate to other regions through inter-regional structural interactions, i.e., individual brain connectome, and produce various ictal symptoms depending on the recruited brain regions.

For the treatment of epilepsy, medication with antiepileptic drugs is preferentially applied [1,2], and surgical intervention is often offered as an option for drug-resistant patients [3–5], which account for more than 30% of patients [2,6]. There are two main types of surgical strategies: resection and disconnection. Resection, which removes the brain regions generating seizures, results in seizure-free outcomes in 30–70% of the postoperative patients depending on the localization accuracy of epileptogenic zone (EZ) and the pathology of each patient [7–10]. Disconnection, which severs nerve pathways that play an important role in seizure propagation, may have either a curative objective, i.e., hemispherotomy, or may limit seizure propagation, i.e., callosotomy [11,12]. Although surgical intervention is generally accepted as an effective method to control drug-resistant seizures, only about 10% of patients might be considered candidates for surgery [6] because EZs are often located in multiple brain regions simultaneously and involve eloquent areas, which are defined as brain regions where damage causes neurological complications such as language, memory and motor problems [13]. Several alternative methods including multiple subpial transection, which could prevent neuronal synchronization in the EZ without altering normal functions by severing horizontal intracortical fibers while preserving vertical fibers in the eloquent cortex, have been tried for patients who are unsuitable for conventional surgery, but with variable results [13–15]. Therefore, there is a clear need to provide more optimal surgical options for those patients. The alternative method must be 1) effective in seizure reduction, 2) able to provide flexible options depending on the inoperable EZ or technically inaccessible region for surgery, and 3) have minimal impact on normal brain functions.

Studies on epilepsy have mainly focused on investigation of the brain network dynamics of individual patients. By analyzing functional data, such as intracranial electrocorticographic (ECoG) signals and stereotactic electroencephalographic (SEEG) signals, many studies have examined network properties for diverse brain states including interictal, preictal, ictal, and postictal epochs [16–21]. In particular, network analysis based on graph theory has been able to not only identify characteristics of the seizure onset zone that would be targeted in resection surgery [17,19], but also observe changes in network topology over the onset and time-course of seizure [22–25]. Several studies have shown that one large regular network is formed at seizure onset compared to the network in the interictal period, which consists of several small sub-networks [22,23]. These results suggest that seizures may be prevented by disrupting the formation of large regular networks through the disconnection of well-chosen sub-networks. Furthermore, several other studies have demonstrated that the epileptic brain network has more segregated features than the healthy brain network [26–28]. Meanwhile, by analyzing structural data based on Magnetic Resonance Imaging (MRI), many studies have reported structural abnormalities in the epileptic brain distinct from the normal brain, which include not only regional alterations (decrease in subcortical volume and cortical gray matter thickness) [29,30], but also abnormalities in white matter tracts, i.e., inter-regional connectivity (reduction in fractional anisotropy) [31,32]. From the network perspective, several studies have shown an increase of local network connectivity and a decrease of global network connectivity in the epileptic brain [27,33,34], even though the situation is more complex depending on whether brain regions are involved in seizures generation and propagation [35,36]. Yasuda and colleagues have further reported that the healthy brain network present widespread distribution of hub regions (frequently used brain regions in inter-regional signaling), while the epileptic brain network has hub regions concentrated in specific areas (for example, in temporal lobe epilepsy, paralimbic/limbic and temporal association cortices) [34]. The results of these studies suggest that the epileptic brain comprises a distinct modular structure and that seizure propagation can be controlled by blocking interactions between the modules, i.e., by severing the connections.

Translation of any computational modeling approach will require the personalization of the brain network models, tailored to a patient’s connectivity and lesion. Personalized brain network models, based on brain connectome and clinical information from each patient, have been able to simulate individual seizure propagation patterns [20,37]. Moreover, some investigations have simulated the effects of surgical intervention, and have been able to predict how the removal of certain brain regions will have an impact on the occurrence seizures [19, 38–42]. These studies show the possibility of computational approaches being able to construct a paradigm that derives optimal surgical strategies for each patient by applying in-silico surgical techniques on the personalized brain network model. However, at present, most efforts in the field focus on improving the localization of EZ and develop strategies to effectively remove the identified zone. Only a few studies have reported the possibility of controlling seizure activities by eliminating the areas other than EZ [40,42].

Here we propose a computational method towards the identification of minimally invasive surgical interventions, particularly applicable for case in which the EZ is non-operable. Focusing on the fact that the epileptic brain network has distinct segregation characteristics, we employ modularity analysis with structural brain connectivity from each patient, in order to derive brain regions and fiber tracts as target zones (TZs) that should be removed for resection and disconnection surgery, respectively. Here, we assume the worst-case scenario in which the EZ is an inoperable zone, so that the proposed in silico surgical approach induces seizure relief by suppressing seizure propagation to other brain areas even though it cannot prevent seizure generation in EZs. Reducing the involvement of propagation networks is a major factor to reduce the impact of seizures, particularly the loss of consciousness [43]. The acquired TZs are evaluated by personalized brain network simulations in terms of the effectiveness to control seizure propagation and the safety to maintain normal brain functions, and then optimized according to the results. The notion of safety is critical to our approach and is here operationalized through network activation paradigms. We leverage our capacity to generate diverse realizations of the same personalized brain model, in particular a healthy and epileptic version. A systematic characterization of the signal transmission characteristics of the healthy brain, here realized via stimulation paradigms, provides us with target templates for the optimization of the safety constraints.

## Results

### In-silico surgical approach

**Fig 1:**
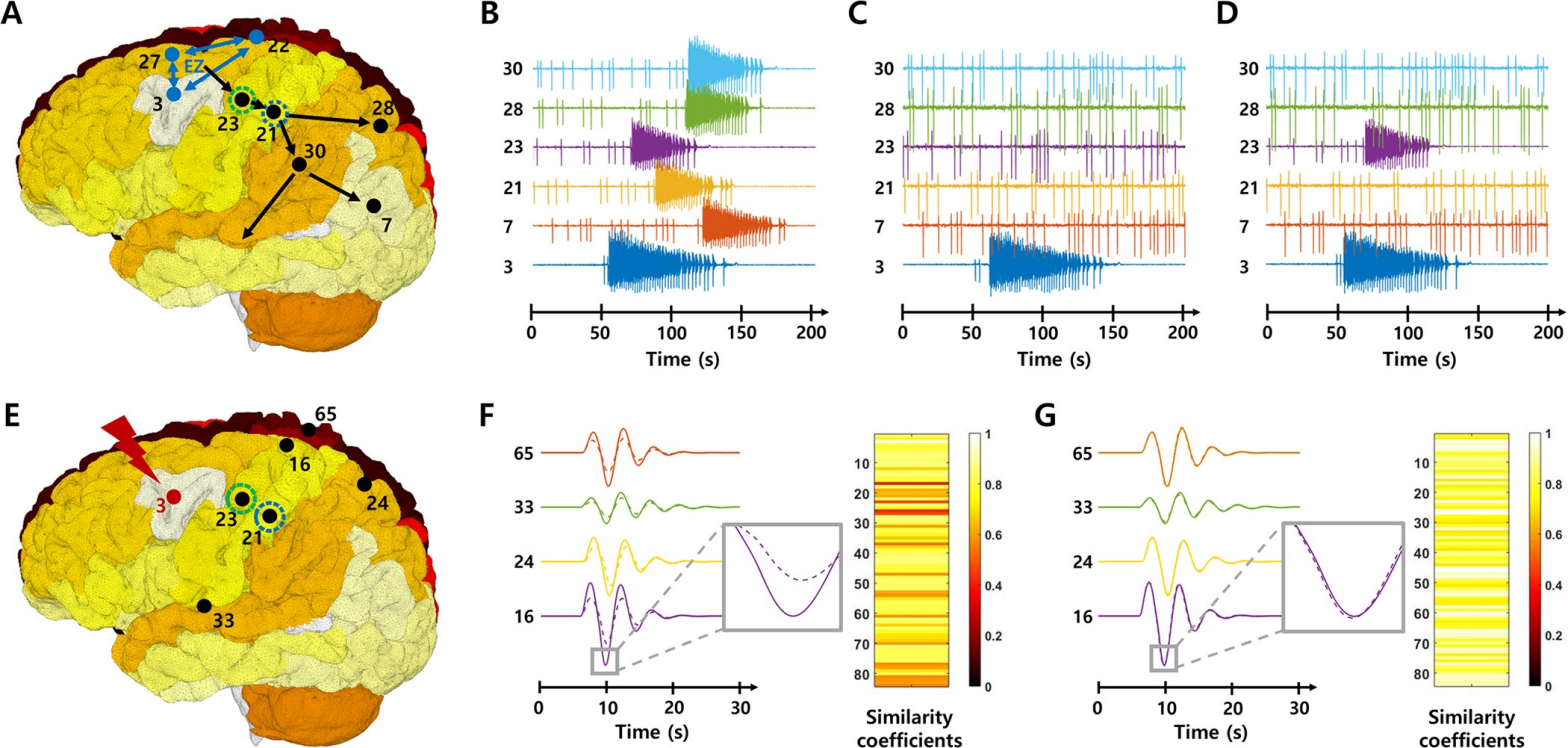
Brain network simulations using TVB. **A-D** Network simulations for the effectiveness evaluation. The brain network model assesses the effectiveness by identifying the seizure propagation characteristics. Figures show simulated signals at each brain node **B** before and **C-D** after removal of the node 23 or node 21 respectively, when nodes 3, 22 and 27 act as EZ. **E-G** network simulations for the safety evaluation. The brain network model assesses the safety by investigating the integrity of the transient spatiotemporal trajectory following electrical stimulation at certain nodes. When a stimulus is applied to the node 3, the stimulation induces different response signals at each node (solid lines, **F-G**). When removing all connections from the node 23, the response signals (dotted lines, **F**) at each node are altered compared to before removal. On the other hand, when eliminating the node 21, the response signals (dotted lines, **G**) are not significantly different from that before removal. The color bars represent similarity coefficients between the response signals at each node before and after elimination.

To derive personalized optimal surgical options for drug-resistant epilepsy patients, we propose a patient-specific in-silico surgical approach combining graph theoretical analysis with brain network simulations. Based on the patient-specific modular structure obtained from the structural brain connectivity and clinical estimation for EZ of each patient, brain regions and fiber tracts acting as hubs in the interaction between the modules, i.e., connecting different modules, are identified as TZs for surgical intervention. The acquired TZs are evaluated through personalized brain network simulations regarding their effectiveness and safety. Fig 1 shows a concrete example for the TZ evaluation.

Effectiveness to control seizure propagation is assessed by the degree of seizure propagation suppression (Fig 1A–1D). Fig 1B presents simulated signals at several brain nodes when nodes 3 (ctx-lh-caudalmiddlefrontal), 22 (ctx-lh-posteriorcingulate) and 27 (ctx-lh-superiorfrontal) are EZs, which show that the nodes are seizure-recruited following some delays depending on connectivity between nodes, after the seizure is generated from EZs. On the other hand, after removing a specific node in the seizure propagation pathway (node 23; ctx-lh-precentral, or node 21; ctx-lh-postcentral), simulated brain signals show that the propagation beyond each node is prevented even if the seizure is still occurred from EZs (Fig 1C and 1D). In this way, characteristics of seizure propagation are observed after eliminating target nodes or target edges for effectiveness evaluation of the TZ. All label names and indices corresponding to the subdivided regions of the brain (brain nodes) are provided in the S1 Table (see the materials and methods section also).

Our approach towards the evaluation of safety of the intervention rests on the maximization of the signal transmission properties of the brain network. The latter is assessed by stimulating relevant brain regions and quantifying the subsequent transient trajectory of brain network activation. More concretely, safety is evaluated by assessing similarity of the spatiotemporal brain activation patterns following electrical stimulation, before and after removal of the TZ. To investigate the variations in resting state (RS) networks, the brain regions where the stimulation is applied are determined based on previous results [44], in which specific brain regions have been reported that can reproduce similar responsive networks to each of the eight well-known RS networks [44,45] (Table 1). Fig 1E–1G present an example of the response network when a stimulus is applied to a specific node (node 3). The stimulation locally activates the stimulated node first, followed by a propagation and sequential recruitment through the connectome, thereby generating a unique spatiotemporal response pattern specific to the stimulation site. The solid lines and dotted lines show the simulated signals obtained from several brain nodes before and after eliminating the certain node, respectively (node 23 in Fig 1F, node 21 in Fig 1G). Compared with the pre-removal response pattern, the response signals are altered following the removal of node 23, whereas the response signals appear unaffected following the removal of node 21. The color bars represent the degree of these differences quantitatively, i.e., as similarity coefficients. These results illustrate nicely the sensitivity of the spatiotemporal seizure organization to network alterations. In this way, the safety of TZ is evaluated by systematically stimulating specific nodes, which reproduce each RS network, and comparing the response patterns before and after eliminating target nodes or target edges. If the TZ is judged to be inadequate based on the network simulation results, another TZ is derived by applying the results to the modularity analysis again. Through this feedback approach, the optimized TZ that effectively prevents seizure propagation while minimally affecting normal brain functions can be obtained. Methodological details for the sequential steps of the proposed in-silico surgical approach are provided in the materials and methods section.

**Table 1:** Stimulation sites to validate RS networks. This table shows stimulus sites able to reproduce the best-matched response patterns with brain activation patterns in each RS network. The number in parentheses indicates the node index.

Resting-state network	Stimulation sites	Resting-state network	Stimulation sites
Default mode (DM)	ctx-lh-medialorbitofrontal (13) ctx-rh-medialorbitofrontal (62) ctx-lh-superiorfrontal (27) ctx-rh-superiorfrontal (76)	Somato-motor (SM) Dorsal attention (DA)	ctx-lh-precentral (23) ctx-rh-precentral (72)
Visual (V) Working memory (WM)	ctx-lh-rostralanteriorcingulate (25) ctx-rh-rostralanteriorcingulate (74)	Memory (M)	ctx-lh-lateraloccipital (10) ctx-rh-lateraloccipital (59)
Auditory-phonological (AP)	ctx-lh-superiortemporal (29) ctx-rh-superiortemporal (78)	Ventral stream (VS)	ctx-lh-caudalanteriorcingulate (2) ctx-rh-caudalanteriorcingulate (51)

### Optimal TZ derivation

Here, we present surgical intervention options outside the EZ, derived from the proposed in-silico surgical approach, for a particular patient (Patient IL). The patient has two EZs, ctx-rh-lingual (node 61) and ctx-rh-parahippocampal (node 64), and these two EZs are designated as inoperable zone.

**Fig 2:**
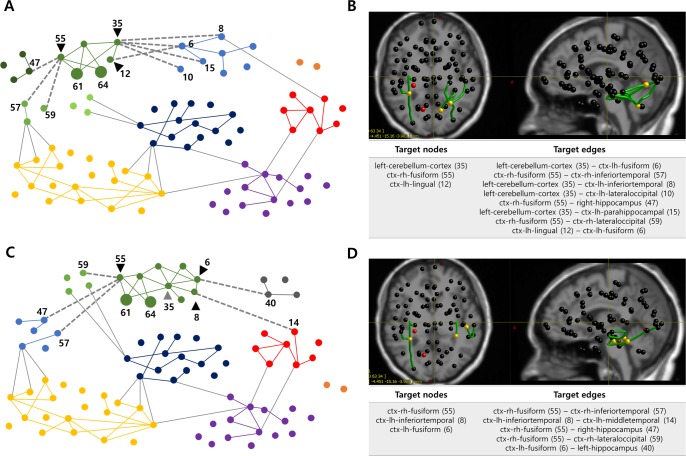
TZs derived from the modularity analysis in Patient IL. **A** Modular structure when setting EZs to inoperable zones (a resolution parameter: 1.25). The brain network is divided into seven modules and the EZ sub-module (green module) is subdivided into four sub-modules, so that each EZ (nodes 61 and 64, large circles) and its neighboring nodes belong to the same sub-module. Based on this modular structure, three nodes (black triangles) and eight edges (gray dotted lines) are derived as target nodes and target edges respectively. For the visualization, only the edges with a connection weight greater than 0.08 are drawn. **B** Anatomical locations and lists of the acquired TZs. Red nodes represent EZs, yellow nodes and green edges indicate target nodes and target edges. **C** Modular structure when adding the critical node (gray triangle) to inoperable zone in modularity analysis (a resolution parameter: 1.25). The brain network is divided into eight modules and the EZ sub-module (green module) is subdivided into two sub-modules, so that each inoperable zone and its neighboring nodes belong to the same sub-module. Based on this modular structure, three nodes (black triangles) and five edges (gray dotted lines) are derived as new target nodes and target edges respectively. **D** Anatomical locations and list of the newly obtained TZs. Red nodes represent EZs, yellow nodes and green edges indicate target nodes and target edges.

Using modularity analysis (see the materials and methods section) we construct a patient-specific modular structure considering inoperable zones (Fig 2A). The brain network nodes are divided into seven modules with modularity coefficient of 0.3912 and the green module including EZs is further subdivided into four sub-modules. Based on this modular structure, 3 target nodes (black triangles) and 8 target edges (gray dotted lines), connecting the EZ sub-module to other sub-modules or modules, are identified. Anatomical location of the initial TZs are shown in Fig 2B.

**Fig 3:**
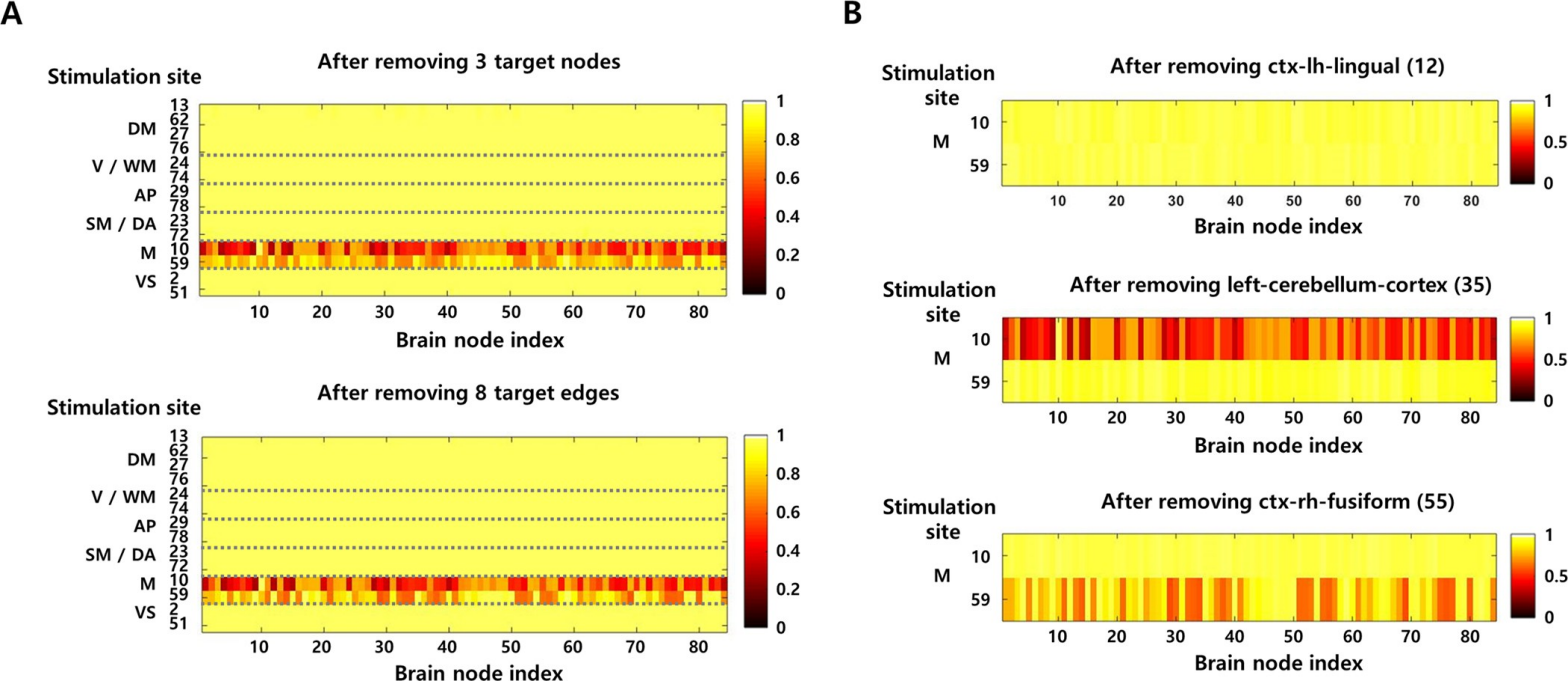
Safety evaluation of the TZs. **A** Network simulation results for safety assessment. When stimulation is applied to the brain nodes that can reproduce each RS network, the difference between response signals before and after removing TZs (3 target nodes or 8 target edges) is presented as the similarity coefficient. The similarity coefficient is calculated independently in all brain nodes, and the color code of the figure indicates the value of the similarity coefficient. Detailed information on stimulation site is provided in the materials and methods section. **B** Identification of the critical node. When each node belonging to the initially obtained target nodes is eliminated, the degree of alteration of the response network (corresponding to the memory network, M) is represented as the similarity coefficient.

In the network simulation for evaluating the effectiveness of the TZs, before the removal of TZs, most brain nodes are recruited after the EZs generate a seizure activity. However, when 3 target nodes are removed, the seizure activity is almost isolated in EZs with a suppression ratio (SR, suppression ratio of seizure propagation) of 95.65%. When 8 target edges are disconnected, seizure-recruited nodes are significantly reduced with the SR of 91.30%, even though the seizure activity is still observed in several neighboring nodes of EZs (results are shown in the S1 Fig). These results demonstrate that the elimination of the derived TZs is able to prevent seizure propagation. Meanwhile, in the network simulation for evaluating the safety of the TZs, similarity coefficients between responsive activation patterns are calculated before and after removal of the TZs, by stimulating specific brain regions to test several RS networks (Fig 3A). Low similarity coefficients indicate that the response pattern due to stimulation has been severely changed after removing the TZs. In this case, the results imply that the elimination of the obtained TZs could lead to a larger network disorganization and then a higher risk for negative cognitive impact, in particular for memory function. Here, the TZs are considered unsafe, if removal of the TZs deforms the response pattern to more than 25% of the original pattern (i.e., if the mean value of similarity coefficients in all brain regions is below 0.75). More details are provided in the materials and methods section.

Since the obtained TZs may have a negative impact on the memory network, the next step is to identify the critical node that leads to the most significant variation. Fig 3B presents the effect on the memory network when each node among the initially derived target nodes is removed. Eliminating the left-cerebellum-cortex (node 35) yields the lowest mean similarity value compared to before removal (0.58, when the stimulus is applied to node 10), so that this node is defined as a critical node, and therefore designated as inoperable zone.

By feeding back the updated inoperable zones to modularity analysis, a new modular structure is obtained. Fig 2C shows the modular structure when the critical node (gray triangle, node 35) as well as two EZs (nodes 61 and 64) are set to inoperable zones. The brain network nodes are divided into eight modules with modularity coefficient of 0.3995, and the green module including EZs is subdivided into two sub-modules so that each inoperable zone and its neighboring nodes belong to the same sub-module. Based on this modular structure, new target nodes (black triangles) and target edges (gray dotted lines) are acquired. Anatomical location of the new TZs are shown in Fig 2D.

**Fig 4:**
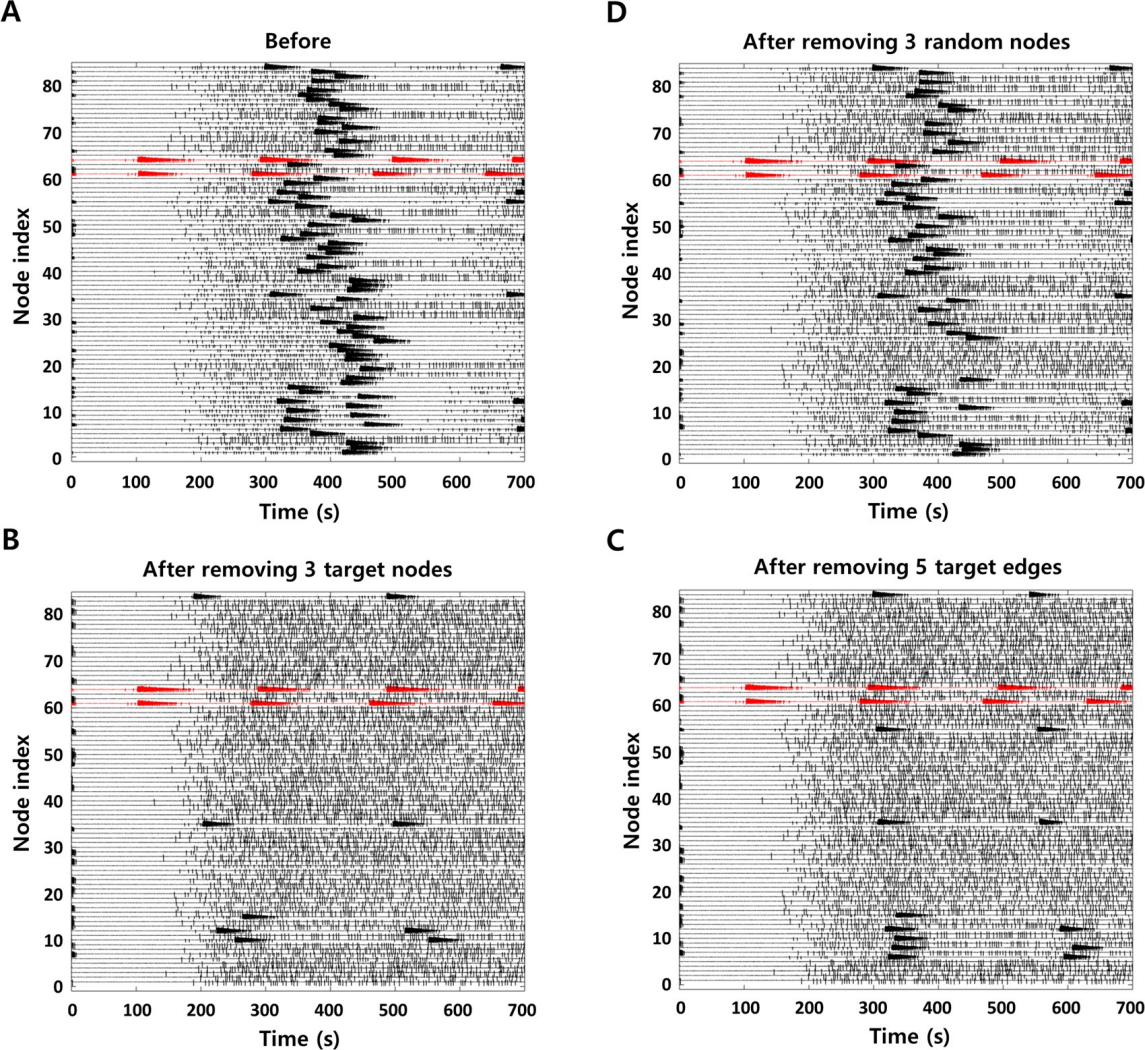
Network simulation results for effectiveness verification. Local field potentials in all brain nodes **A** before removing TZs **B** after removing 3 target nodes **C** after removing 5 target edges **D** after removing 3 random nodes. The figures show propagation characteristics of the seizure occurred from EZs (node 61 and 64, reds).

Fig 4 shows network simulation results for effectiveness evaluation of newly derived TZs. The results present time series data, i.e., local field potentials, in all brain nodes. Before the removal of TZs, the seizure activity originated from EZs propagates to other nodes after some delay, i.e., most nodes are seizure-recruited (Fig 4A). Having eliminated the new TZs, a significant reduction of seizure-recruited regions is identified compared to pre-removal simulation (Fig 4B and 4C; the SR after removing 3 new target nodes: 89.86%, the SR after removing 5 new target edges: 85.51%), even though they have some more seizure-recruited regions than when removing initial TZs. Fig 4D shows the simulation results when removing the same number of random nodes (excluding EZs) as the derived target nodes. Comparing the degree of reduction in seizure-recruited nodes, it demonstrates that the elimination of TZs obtained from the proposed method can effectively suppress the seizure propagation (in this example, the SR after removing 3 random nodes: 31.88%). Meanwhile, the simulation results show that persistent spikes occur even if seizure activity is suppressed in each brain node after the removal of TZ. These interictal spikes are caused by the noise environment that we apply for stochastic simulations. In this study, Gaussian noise is applied to all brain nodes (Epileptors) to account for background internal activity, so that each node generates random spike events as a baseline activity. The occurrence of these spikes is regulated according to the state of each node, such as preictal, ictal and postictal. Methodological details are provided in the materials and methods section, and more details on the behavior of the Epileptor model can be found in previous papers [37,46].

**Fig 5:**
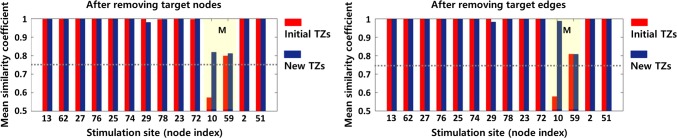
Safety evaluation results for the initial TZs and the new TZs obtained by the feedback. The histogram shows the mean value of the similarity coefficients between the responsive activation patterns due to stimulation in all brain regions, before and after removal of the target nodes and target edges, respectively. While the elimination of initial TZs has a value lower than threshold (0.75) when stimulation is applied to node 10 to reproduce memory network (M), the removal of new TZs has values higher than the threshold in all stimulation sites.

Fig 5 shows the difference between the safety evaluation results for initial TZs and new TZs. The histogram shows the mean value of the similarity coefficients between the response patterns due to stimulation in all brain regions before and after removal of TZs. Comparing the values between two groups, it indicates that eliminating the new TZs is able to maintain all RS networks at a similar level as before removal (the mean value of similarity coefficients > 0.75), whereas eliminating the initial TZs may disrupt memory network. In other words, this means that the newly derived TZs have less impact on the transmission properties of the brain network sustaining normal brain function. The results also show that disconnecting the fiber tracts corresponding to the target edges has less impact on normal brain function than resecting the brain regions (corresponding to the target nodes). In this example, the new TZs obtained from a single feedback satisfy the safety criteria. However, if the newly derived TZs do not satisfy the criteria, the iterative feedback procedure (find a critical node among the new TZs, set it to inoperable zone, and obtain a new modular structure) continues until the TZs that meet the criteria are derived.

In this section, we present the results for condition when the resolution parameter in the modularity analysis is fixed to 1.25 (from Fig 2 to Fig 5) for simplicity. However, since the proposed method involves a parameter sweep of the resolution parameter (0.5 to 1.5 with intervals of 0.25), multiple modular structures are obtained according to the parameter value (the resolution parameter determines the size of each module, i.e., the number of modules), resulting in multiple TZs options. In this patient, 5 variants for target node and 7 variants for target edge have initially been obtained. After applying the feedback, 7 variants for target node and 9 variants for target edge have finally been derived. The lists of TZ variants are presented in S2 Table. Results for 6 other patients are also shown in S3–S8 Tables and S3–S8 Figs. The results contain several TZ variants that are appropriate each patient’s circumstance considering the location of EZ and individual brain connectome. The final TZ variants should be effective surgical targets preventing seizure propagation with maintaining normal brain functions.

### Systematic analysis according to EZ location

**Fig 6:**
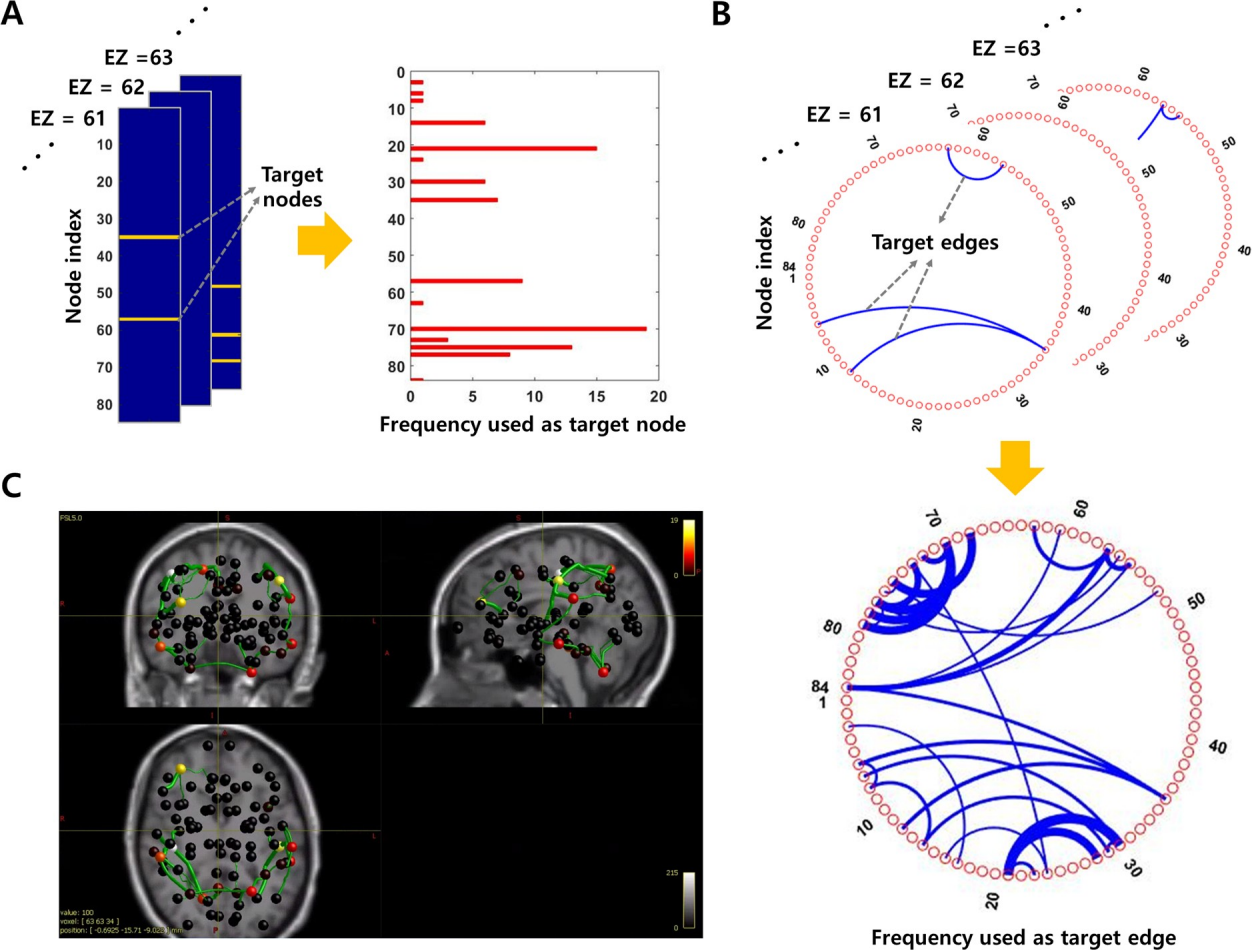
TZs depending on the location of EZ. **A** Target nodes according to the location of EZ and their cumulative results. Orange points in each column indicate the target nodes, when EZ is located in each different node. **B** Target edges according to the location of EZ and their cumulative results. Connection lines between nodes in each slice indicate the target edges, when EZ is located in each different node. The cumulative result identifies several nodes and edges frequently used as TZ. Here, the resolution parameter for the modularity analysis is set to 1.0. **C** Anatomical locations of the nodes and the edges frequently obtained as TZ. The color code of nodes and the thickness of edges indicate the frequency used as TZ.

Here, in order to demonstrate the robustness of the proposed method, we present additional simulation results that show how TZ varies according to the location of EZ. Fig 6A and 6B show target nodes and target edges, in a specific patient (Patient CV), obtained by performing systematic simulations where one EZ is placed in all possible brain nodes (The EZ is assumed to be an inoperable zone). The cumulative results of TZs identify the nodes and the edges that are frequently used as TZs. Frequently acquired nodes and edges play an important role in propagating seizure activity from the localized region to the entire brain, and can effectively control seizure propagation by being removed. In this patient, the most frequently derived node is ctx-rh-postcentral (node 70), and the most frequently derived edge is the connection between ctx-lh-supramarginal (node 30) and ctx-lh-postcentral (node 21). Anatomical locations of cumulative results are presented in Fig 6C. Meanwhile, in deriving the TZs, the frequency of the target nodes initially acquired is positively correlated with the node strength (the sum of weights of links connected with other nodes), i.e., the nodes having high strength are frequently derived as TZs (correlation coefficient: 0.7842). However, the final target nodes obtained from the feedback procedure tend to be more concentrated at few nodes, and thus the frequency of the finally acquired target nodes is not noticeably relevant to the node strength (correlation coefficient: 0.3059). Simulation results for 6 other patients are shown in S9–S14 Figs.

Interestingly, the critical nodes, which are used for the feedback strategy to consider the safety for normal brain functions, are not significantly different in all 7 patients. In particular, the superior-frontal cortex (nodes 27 and 76) appears often as the critical node, which means that these nodes are effective to control seizure propagation but removing them may cause a problem for the normal brain function (this region is also the node with the highest strength). The network simulation results identify that the elimination of those nodes severely distort the RS networks corresponding to visual, working memory and ventral stream as well as default mode. In fact, in previous studies, the superior-frontal cortex has been investigated as a node that is frequently used as the shortest path connecting two different brain nodes [47], and also has been shown to play an important role in interhemispheric propagation of seizures [41]. Furthermore, several clinical studies have reported the resective surgery in the superior-frontal cortex, which indicate that it may cause working memory impairment [48] as well as transient motor deficit [49,50].

In this section, the systematic simulations have been demonstrated for different TZs according to the locations of EZ. The results can be used not only to identify major nodes and edges involved in seizure propagation, but also as a reference to elicit reasonable surgical targets if there are several clinical hypotheses for the EZ location.

## Discussion

We have demonstrated the use of personalized brain network models for the development of novel surgical intervention. In particular, we focused on deriving effective alternative methods for those cases where EZs are inoperable, so that we conducted the study assuming that all EZs of patients are inoperable even if some EZs are clinically removable. Our proposed in-silico surgical approach is based on the graph theoretical analysis using patient-specific brain connectome, specifically modularity analysis, and personalized brain network simulations. We propose a strategy to operationalize the notion of “safety” by minimizing the impact upon the brain’s signal transmission capacity.

The modularity analysis is generally used as method to investigate synchronization characteristics between brain regions [23,33,51,52]. Consistent with previous observations [26,33,34], we found that each patient's brain network has a distinct modular structure. From the patient-specific modular structure, nodes and edges connecting the EZ sub-module with other submodules or modules were extracted as surgical options, TZs, to suppress seizure propagation in a patient-specific manner. By adding a constraint to the existing modularity analysis, flexible TZs excluding inoperable zones could be derived, which may provide alternative surgical methods that can result in seizure relief to patients who are considered unsuitable for the conventional surgery since resection of EZ may cause severe neurological complications. Moreover, the parameter sweep in the modularity analysis obtained different modular structures, ultimately resulted in multiple TZ options. This multiplicity is crucial in that clinicians can select the surgical target within multiple options, taking into account the number of interventions and the suppression degree of seizures. Clinicians may also consider not only the specific regions that should be excluded for surgery based on their clinical experiences but also the technically challenging regions.

To evaluate the effectiveness and safety of the identified TZ, brain network simulations were employed. Based on the patient-specific network model constructed by structural brain connectivity and clinical estimation for EZs of each patient, the effectiveness of the TZs were assessed by simulating seizure propagation characteristics before and after removal of the TZs. Reducing the involvement of propagation networks is a major factor to reduce the impact of seizures, particularly the loss of consciousness [43]. Loss of consciousness is one of the major signs and is clearly linked to the synchronization in propagation network, particularly fronto-parietal networks during temporal lobe epilepsy (TLE) seizures. It is recognized that a good outcome after epilepsy surgery (according to the Engel's classification) may include patients with residual subjective symptoms (aura) but without any more objective signs (automatism, loss of consciousness), which is the definition of seizure free patients IB in Engel's classification.

In the literature, other in-silico surgical approaches have been reported recently [39,41]. Hutchings and colleagues have identified seizure reduction by detecting and eliminating nodes having a fast transition time to seizure state through network simulations, which was constructed using the structural connectivity of each patient [41]. Sinha and colleagues have proposed a network model, based on functional brain connectivity of each patient, which has predicted the regions having higher likelihood of seizure occurrence [39]. They have shown that better surgical outcomes can be produced if the actual surgical site well matches the region obtained from the simulation [39]. In contrast to our approach, most previous studies have focused on decrease of seizure occurrence by removing the brain regions where seizures occur first, i.e., seizure onset zones. These approaches of no use when the regions in question are inoperable and alternative approaches, as advocated here, need to be considered imperatively. Meanwhile, a few recent in-silico studies have reported the effects of resection of non-EZ areas on the epileptic networks [40,42]. The studies have demonstrated that eliminating a node other than the hyperexcitable node can be effective to reduce the seizure occurrence as well as removing the hyperexcitable node (or, it may be more effective [40]), so that they have shown the possibility of a computational framework to suggest alternative surgical strategies. However, since the effect on seizure reduction is highly dependent on the location or contribution of each node in the network, a more systematic approach is needed to identify the target node (i.e., alternative surgical target). Furthermore, network effects should be investigated at a whole-brain scale. Here, we have derived TZs based on the clinical estimation and brain connectivity analysis of each patient, and examined the effect of TZ removal in the seizure propagation network through personalized brain network simulation based on individual brain connectome.

Critical to surgical intervention outside of the EZ is the investigation of the safety of the procedure. We here operationalized safety by the concept of preservation of signal transmission properties of the brain network, assuming those to be directly linked to brain function. Brain function capacity is often, at least implicitly, quantified by functional connectivity of the resting state [53–59]. These approaches attempt to quantify, by construction, properties of attractor states at rest. Several computational studies have simulated resting state and task-related functional connectivity (RS-FC) through a large-scale brain network model, and shown the correlation with empirical human brain imaging data including functional MRI (fMRI) signals [53,58,59]. However, considering the variability of RS-FC observed in both empirical and simulation data [58], these approaches may not be sufficient to compare the effects before and after removal of a specific brain region (or a specific connection). Therefore, a straightforward method is required to evaluate the TZs by distinctly quantifying the changes in network characteristics at resting state in pre- and post-surgical condition. Perturbations to attractor states allow to sample additional properties of the brain network such as attractor stability, convergence and divergence of flows, and thus significantly enhance the characterization of its dynamic properties. Stimulation is a simple but reliable way to induce perturbation to each state, which generates a spatiotemporal response pattern according to the stimulation location and brain connectivity. Here, we employed the stimulation method to reproduce each RS network and to clearly quantify the changes in the network properties before and after eliminating the TZs. To best estimate the transient spatiotemporal trajectory due to stimulation applied to individual brain regions, we compared its spatial and temporal properties before and after eliminating the TZs. Analyses of this nature have been performed previously by Spiegler et al [44] and demonstrated that transient trajectories are highly constrained by the structural properties of the network and show a surprisingly low-dimensional behavior, after an initial local stimulation artifact. Here we have exploited these transient trajectory properties to quantify the difference of response network due to stimulation, and assumed that the changes in the response pattern after removal of TZ indicate a negative impact in terms of brain functionality (i.e., we have interpreted the TZ as unsafe if the difference in response patterns before and after removal of the TZ is large). However, some clinical studies have reported postoperative cognitive improvements in epilepsy patients [60,61]. In particular, Baxendale and colleagues have demonstrated improvements in memory function (verbal learning and visual learning) in about 10% to 20% of patients who underwent anterior temporal lobe resection [60]. These results indicate that, in contrast to the assumption we made in this paper, changes in the response pattern after elimination of TZs may have a positive impact on the functionality. This limitation should be sufficiently discussed and improved by the integrated and parallel approaches with clinical studies.

In clinical routine, pre-surgical mapping of eloquent cortex is routinely conducted [62–64] including electrical stimulation through implanted electrodes [62]. These mappings make it possible to identify important cortical regions that should be excluded from the surgery because they are most disruptive. As noninvasive methods, fMRI and Magnetoencephalography (MEG) are frequently used for the mapping [63,64], they localize eloquent cortex by identifying activated regions during certain tasks, such as motor, memory and language functions [64]. Despite these efforts, current epilepsy surgery still results in transient and permanent neurological complications including visual field defects, memory disturbances, dysphasia and hemiparesis [65–67]. In fact, except for the primary cortices responsible for specific functions, there is still a limit to accurately predicting what deficits may result from the removal of a specific brain region. Especially, with respect to RS networks, despite the fact that it is necessary to minimize postoperative changes in the functional networks, there is no index that can systematically evaluate the changes. Furthermore, the reported morbidity rates show a large variability across institutions [65,67], suggesting that surgical outcomes, including postoperative deficits, are highly dependent on diagnostic procedures and decisions about the surgical planning. This observation is also known from a variety of other decision making situations including medicine and economics, best paraphrased by Daniel Kahneman, *“To maximize predictive accuracy*, *final decisions should be left to algorithms*, *especially in low-validity environments”* [68]. Taking into account these current limitations related to the prediction of postoperative deficits, we emphasize that the use of more systematic and integrative methods is *condition sine qua non* to quantitatively predict the impact of the removal of a specific region on normal brain functions. The computational method based on personalized brain network modeling suggests a novel approach to evaluate the safety of the surgery, and can be enhanced by combining with conventional clinical methods (such as task fMRI and RS fMRI).

Our proposed in-silico surgical approach derives personalized optimal TZs considering inoperable zones, by means of a feedback approach combining modularity analysis and brain network simulations. However, given that our current work addresses network modulation outside of the EZ, it has challenges linked to clinical validation, because it is by definition outside the clinical routine. Extensive clinical data sets that have undergone surgery for areas other than EZ, in particular disconnection surgeries such as partial hemispherotomy [69], can be used to validate the proposed method. Individualized modeling for each data set and simulation results reflecting the actual surgical site can be directly compared with post-operative clinical outcomes (empirical data). Animal experimental models that can reproduce relatively diverse protocols can further test the network modulation results of this study. Although validation limits remain, the here presented in-silico analyses open new grounds for the discovery of novel surgical interventions.

The proposed method has also some limitations in terms of the brain network model. First, in this study, to verify the effectiveness of TZs, we used a constant excitability parameter value for all other brain regions excluding EZs in order to assume the worst-case scenario, i.e., we used a relatively higher value corresponding to the PZ for all other regions. However, each brain region has different excitabilities in real world systems, and PZs could be limited to only a few regions, even though it depends on the brain connectivity and the number and location of EZs in each patient [20,37]. In particular, through the in-silico surgical approach, the cerebellum was sometimes derived as a TZ when the same excitability value as other brain regions was applied because it has strong connectivity with other brain regions (i.e., it plays important role in seizure propagation). However, in the real world system, the cerebellum has a low excitability so seizure recruitment rarely occurs. We have not considered this regional specificity in our virtualizations so far, but detailed atlases and integration with The Virtual Brain (TVB) platform will enable this line of further improvement. Secondly, we divided the patient’s brain network into 84 regions, and modeled each region as one node. Each brain region was connected through structural connectivity, so it was reasonable to observe the propagation characteristics of seizure generated from certain regions. However, since neurophysiological mechanisms including interactions between neurons within a specific region, i.e., internal connections, was not reflected, high-resolution spatial synchronization phenomena could not be observed. This issue can be improved through further studies, which model each brain region into multiple nodes and define internal connections that represent interactions between the nodes within a region. Despite some limitations, our study has a great importance in that it demonstrates that computational approaches pave the way for personalized medicine, by deriving innovative surgical options suitable for each patient and predicting the surgical outcomes.

## Materials and methods

The personalized in-silico surgical approach was based on graph theoretical analysis and brain network simulations. Preferentially, from the modularity analysis considering inoperable zones, brain regions and fiber tracts acting as hubs in the interaction between the modules were derived as TZs. Then, the obtained TZs were evaluated in terms of the effectiveness and the safety by personalized brain network simulations using TVB, a platform to simulate the brain network dynamics [70]. If the TZ did not satisfy the evaluation criteria, a new TZ was derived by feeding back the simulation results to the modularity analysis again. Through the feedback approach, the optimized TZ options that minimize seizure propagation while not affecting normal brain functions could be obtained. A detailed description of each step is provided below.

### Structural brain network reconstruction

Neuroimaging data was obtained from 7 drug-resistant epilepsy patients. The patients had EZs with different locations and underwent comprehensive presurgical evaluations [37,38]. The clinical characteristics of each patient are provided in S9 Table [37]. The structural brain network of each patient was reconstructed from diffusion MRI scans and T1-weighted images (Siemens Magnetom Verio 3 T MRscanner) using SCRIPTS pipeline [20,37,71]. Each patient’s brain was divided into 84 regions, which included 68 cortical regions based on the Desikan-Killiany atlas [72], and 16 subcortical regions (all label names for the subdivided regions are listed in the S1 Table). Connection strengths between the brain regions were defined based on the number of streamlines (fiber tracts), and tract lengths to determine signal transmission delays between the regions were also derived.

### TZ derivation based on the patient specific modular structure

#### Modularity analysis

To analyze the modular structure of the brain network, we used a previously reported Matlab toolbox [73]. The modularity analysis based on Newman’s spectral algorithm provided the non-overlapping modular structure that minimize edges between modules and maximize edges within modules [74]. It computed the leading eigen vector of the modularity matrix *B* (Eq (1)) and divided the network nodes into two modules according to the signs of the elements in the eigen vector. In the equation, *A*_*ij*_ represents a weight value between node *i* and node *j*, *k*_*i*_ and *k*_*j*_ indicate the degree of each node, *m* denotes the total number of edges in the network. *α* is a resolution parameter for the analysis, the classic value is 1. Then, the division was fine-tuned by the node moving method to obtain maximal modularity coefficient Q. The modularity coefficient has a value ranging from 0 to 1, the value of 0.3 or higher generally indicates a good division [75]. *s*_*i*_ and *s*_*j*_ represent group membership variables that have a value of +1 or -1 depending on the group to which each node belongs. Each module, which was divided based on the eigen vector algorithm, was further divided into two modules until there is no effective division that results in a positive modularity coefficient.

$$B_{ij}=\left(A_{ij}-\alpha \frac{k_{i}k_{j}}{2m}\right),\;Q=\frac{1}{4m}\sum_{ij}B_{ij}s_{i}s_{j}$$ (1)

In this study, we added a constraint to the existing toolbox in order to prevent inoperable nodes from being derived as TZ. First, the group membership variable values of the nodes classified by the eigenvector algorithm are identified. Then, if the inoperable node and its neighboring nodes (adjacent nodes based on the weight matrix) did not have the same value, it set the values of them to the value that most of them have. In other words, the constraint limits the inoperable node and its neighbor nodes to belong to the same module, so that the inoperable node does not act as a hub connecting the modules. Meanwhile, the resolution parameter *α* was swept from 0.5 to 1.5 with intervals of 0.25 to obtain multiple modular structures. The resolution parameter determines the size of each module, i.e., the number of modules, in dividing the network nodes into modules. A high parameter value derives a modular structure consisting of small modules (i.e. the large number of modules) and a low parameter value obtains a structure consisting1 of large modules (i.e., the small number of modules).

#### Target node and target edge

To derive TZ from the modularity analysis, EZ and inoperable zone should be set first. The EZs were fixed according to the clinical evaluation of each patient, and the inoperable zones were arbitrarily set to all EZs, i.e., we assumed the worst-case scenario in which all EZs cannot be surgically removed. In detail, we wanted to obtain TZs excluding all EZs for resection surgery and excluding all fiber tracts connected to the EZs for disconnection surgery.

Our strategy to suppress the seizure propagation is to divide each patient's brain network into multiple modules and then remove the connections (nodes or edges) from the module containing the EZ (EZ module) to the other modules. However, in the modularity analysis, when a low resolution parameter is used, a relatively large number of nodes may belong to the same module with EZ, and eventually, still quite a few nodes may be seizure-recruited even if the TZs are eliminated. To control this issue (i.e., to prevent a significant number of nodes from becoming seizure-remained nodes), we chose a strategy to divide EZ module to sub-modules once again and define the TZs as the nodes/edges that connect the submodule including the EZ (EZ submodule) to other submodules or modules. We named the nodes and the edges acquired for resection and disconnection surgery as target nodes and target edges, respectively.

Since we controlled the resolution parameter in the modularity analysis (in both division processes), multiple modular structures were obtained for the same patient, thereby it could provide multiple intervention options for target nodes and target edges. All of the procedures described above were automatically performed by the Matlab model that we have developed. The model could yield multiple TZ options according to the location of EZ and inoperable zone.

### Brain network simulation using The Virtual Brain

#### Effectiveness evaluation

Patient-specific network models were constructed using TVB in order to verify the effectiveness of derived TZs. The six-dimensional Epileptor model was specifically employed to describe a network node and the reconstructed structural connectivity was used to connect the nodes. The Epileptor is a phenomenological neural population model reproducing seizure characteristics [20,46], which consists of five state variables and six parameters (Eq (2)). Each Epileptor was coupled with others via the permittivity coupling of slow time scales variable z replicating extracellular effects [76]. In the equation, *K*_*ij*_ denotes the connection weight between node *i* and node *j*, and *τ*_*ij*_ represents the time delay determined by track length between the two nodes [20,37,76].

$$\begin{array}{l} {\dot{x}}_{1,i}=y_{1,i}-f_{1}(x_{1,i},x_{2,i})-z_{i}+I_{1,i}\\ {\dot{y}}_{1,i}=1-5{\left(x_{1,i}\right)}^{2}-y_{1,i}\\ {\dot{z}}_{i}=\frac{1}{\tau_{0}}\left\{4\left(x_{1,i}-x_{0,i}\right)-z_{i}-\sum_{j=1}^{N}K_{ij}(x_{1,j}(t-\tau_{ij})-x_{1,i})\right\}\\ {\dot{x}}_{2,i}=-y_{2,i}+x_{2,i}-{\left(x_{2,i}\right)}^{3}+I_{2,i}+0.002g(x_{1,i})-0.3(z_{i}-3.5)\\ {\dot{y}}_{2,i}=\frac{1}{\tau_{2}}\left\{-y_{2,i}+f_{2}(x_{2,i})\right\} \end{array}$$ (2)

Where

$$\begin{array}{l} f_{1}(x_{1,i},x_{2,i})=\left\{ \begin{array}{l} {x_{1,i}}^{3}-3{x_{1,i}}^{2}\;if\;x_{1,i}<0\\ \{x_{2,i}-0.6{\left(z_{i}-4\right)}^{2}\}x_{1,i}\;if\;x_{1,i}\geq 0 \end{array}\right.\\ f_{2}(x_{2,i})=\left\{ \begin{array}{l} 0\;if\;x_{2,i}<-0.25\\ 6(x_{2,i}+0.25)\;if\;x_{2,i}\geq -0.25 \end{array}\right.\\ g(x_{1,i})=\int_{t_{0}}^{t}e^{-\gamma (t-\tau )}x_{1,i}(\tau )d\tau \end{array}$$

Clinically, degrees of epileptogenicity may be mapped upon the excitability parameter *x*_0_ where we distinguish EZ that generates spontaneous seizure activities, propagation zone (PZ) that is recruited by seizure propagation from EZ, and other zones not recruited in the propagation [20]. In this study, we set the excitability parameter *x*_0_ to -1.6 for EZ, and a value between -2.150 and -2.095 corresponding to PZ for the all other nodes depending on structural connectivity of each patient, in order to simulate the worst-case scenario at which seizure activity originated from EZ propagates to most other brain nodes. For the other parameters in the equations, we used *I*_1_ = 3.1, *I*_2_ = 0.45, *γ* = 0.01, *τ*_0_ = 6667 and *τ*_2_ = 10. Also, zero mean white Gaussian noise with a standard deviation of 0.0003 was linearly added to the variables *x*_2_ and *y*_2_ in each Epileptor for stochastic simulations. These noise environments made each Epileptor excitable and thus produced interictal spikes, as a baseline activity.

Using the patient-specific network model, we simulated the seizure propagation characteristics before and after eliminating target nodes or target edges. In particular, we quantified the suppression ratio of seizure propagation as Eq (3) and used it to compare the removal effect of each TZ. *x*_1_+*x*_2_ waveform of each Epileptor was observed to reproduce local field potential at each node.

$$\begin{array}{c} \mathrm{SR},\mathrm{suppression}\;\mathrm{ratio}\;\mathrm{of}\;\mathrm{sezure}\;\mathrm{propagation}=\left(\frac{N_{bef}-N_{af}}{N_{bef}}\right)*100\;\left(\%\right),\\ N_{bef}=the\;number\;of\;seizure‐recruited\;nodes\;before\;removal\;of\;TZs,\\ N_{af}=the\;number\;of\;seizure‐recruited\;nodes\;after\;removal\;of\;TZs \end{array}$$ (3)

#### Safety evaluation

To assess normal brain function we adapted a stimulation paradigm, in which we quantified the information transmission capacity of the network through the spatiotemporal properties of the trajectory leading to its resting state, after a transient stimulation. Eight particular well-known RS networks were tested, which include default mode, visual, auditory-phonological, somato-motor, memory, ventral stream, dorsal attention and working memory [44,45]. Previous work has shown that stimulating a specific brain region could reproduce dynamically responsive networks similar to brain activation patterns in RS networks [44]. Spiegler and colleagues have reported the best matched stimulation sites with each RS network in cortical and subcortical regions [44].

Based on the previous studies, we chose to apply an electrical pulse of 2.5s to a particular cortical region and observed the response signals in all brain regions. The stimulation sites to test each RS network are shown in Table 1, the number in parentheses represents the node index. In this simulation, we used the patient-specific network models as before, with the neural mass model of the generic 2-dimensional oscillator (Eq (4)) rather than the Epileptor, in order to replicate damped oscillations due to the stimulation (see [44]). For the parameters, we used *τ* = 1, *a* = −0.5, *b* = −15.0, *c* = 0.0, *d* = 0.02, *e* = 3.0, *f* = 1.0 and *g* = 0.0. Each oscillator was coupled with other oscillators via difference coupling based on individual structural brain connectivity. Here, each oscillator (brain node) operated at a stable focus in proximity to the instability point, supercritical Andronov-Hopf bifurcation, but never reached the critical point. Each node showed no activity without stimulation, but when stimulated (or received input from other nodes through connectome), it generated a damped oscillation by operating closer to the critical point. Since the working distance to the critical point was determined depending on each node’s connectivity (connection weights and time delays), each node generated different damped oscillations (with different amplitudes and decay times), thereby producing a specific energy dissipation pattern (responsive activation pattern) according to the stimulation location and brain connectivity.

$$\begin{array}{c} \dot{v_{i}}=d\tau \;(-f{v_{i}}^{3}+e{v_{i}}^{2}+gv_{i}+w_{i}+\sum_{j=1}^{N}K_{ij}(v_{i}(t-\tau_{ij})-v_{i}))\\ \dot{w_{i}}=\frac{d}{\tau }(c{v_{i}}^{2}+bv_{i}-w_{i}+a) \end{array}$$ (4)

Then, we compared the responsive spatiotemporal activation patterns before and after removing target nodes or target edges. To do so, we quantified the subspace, in which a trajectory evolves after stimulation, by employing mode level cognitive subtraction (MLCS) analysis [77]. From the principal component analysis (PCA) using response signals in all brain nodes before in-silico surgery, reference coordinate system was derived, i.e., eigenvectors *φ*_*n*_ of covariance matrix of response signals were calculated. Then, three principal components (PC) were selected and response signals in both cases (before and after removal of TZ, *q*_*b*_, *q*_*a*_) were projected upon the PC, reconstructed responsive signals *q*_*r*,*b*_, *q*_*r*,*a*_ were obtained at each brain node (Eq (5)).

$$\begin{array}{c} q_{r,b}=\sum_{n=1}^{3}{\varphi_{n}\eta }_{n,b}(t),\;\eta_{n,b}(t)={\varphi_{n}}^{T}q_{b}\\ q_{r,a}=\sum_{n=1}^{3}{\varphi_{n}\eta }_{n,a}(t),\;\eta_{n,a}(t)={\varphi_{n}}^{T}q_{a} \end{array}$$ (5)

To compare the reconstructed responsive patterns, we calculated the amount of overlap between the powers of the reconstructed response signals before and after eliminating TZ, for every brain node. The obtained value in each brain node was normalized by the overlap value using only the signal power before removal of TZ, and then defined as the similarity coefficient (defined as 1−the deviation from 1, if the value > 1; thereby, the similarity coefficient has a value between 0 and 1). Here, we considered that the derived TZ has a high risk if the mean value of similarity coefficients in all brain regions is below 0.75. In other words, it indicates that the elimination of the TZ could affect the corresponding RS network. We referred to the TZ with high risk as inoperable zone. If the TZs contained more than one node, we figured out the critical node that severely changed the responsive activation patterns due to stimulation, and then designated that node as inoperable zone. The critical node was defined as a node that yielded the lowest similarity coefficients when the same simulation was repeated after removing each node belonging to the TZ. The updated inoperable zone (added the critical node) was applied to the modularity analysis again, which resulted in a new TZ. The effectiveness and safety of the newly obtained TZ were evaluated through network simulations again. These feedback procedures were iterated until the TZs that meets the safety criteria were acquired.

## Supporting information

S1 Table Labels and indices of sub-divided brain regions. (DOCX)

S2 Table TZ variants obtained from the proposed in-silico surgical approach (Patient IL). (DOCX)

S3 Table TZ variants obtained from the proposed in-silico surgical approach (Patient CJ). (DOCX)

S4 Table TZ variants obtained from the proposed in-silico surgical approach (Patient FB7). (DOCX)

S5 Table TZ variants obtained from the proposed in-silico surgical approach (Patient FB6). (DOCX)

S6 Table TZ variants obtained from the proposed in-silico surgical approach (Patient PC). (DOCX)

S7 Table TZ variants obtained from the proposed in-silico surgical approach (Patient CV). (DOCX)

S8 Table TZ variants obtained from the proposed in-silico surgical approach (Patient SF). (DOCX)

S9 Table Clinical characteristics of patients. Th, thermocoagulation; Gk, Gamma knife; Sr, surgical resection; NO, not operated; N, normal; FCD, focal cortical dysplasia; SPC, superior parietal cortex; Fr, Frontal; PVH, periventricular nodular heterotopia; NA, not available; L, left; R, right (From [37]).
(DOCX)

S1 Fig Network simulation results for effectiveness verification of initial TZs (Patient IL). The figures show propagation characteristics of the seizure occurred from EZs (node 61 and 64) before (left) and after eliminating target nodes (middle) or target edges (right).
(TIF)

S2 Fig Anatomical locations and simulation results for the optimal TZ (Patient IL). **A** Anatomical locations of the TZs. Red nodes represent EZs (61, 64), yellow node (6, 8, 55) and green edge (6–40, 55–59, 55–47, 8–14, 55–57) indicate target node and target edge. **B** Network simulation results for safety verification. The results show the similarity coefficients between responsive activation patterns due to electrical stimulation before and after removing TZs. **C** Network simulation results for effectiveness verification. Before removing TZs, the seizure activity occurred from EZs (61, 64) propagates to most brain regions after some delays (left). By eliminating the target node (middle) or the target edge (right), seizure recruited regions are decreased significantly, even though the EZ generate seizure activities continuously.
(TIF)

S3 Fig Anatomical locations and simulation results for the optimal TZ (Patient CJ). **A** Anatomical locations of the TZ. Red nodes represent the EZ (10), green edges (35–61, 6–12, 7–30, 14–29, 35–12, 7–28, 35–84) indicate target edges. In this case, effective and safe target nodes for resection surgery are not obtained. **B** Network simulation results for safety verification. The results show the similarity coefficients between responsive activation patterns due to electrical stimulation before and after removing TZs. **C** Network simulation results for effectiveness verification. Before removing TZs, the seizure activity occurred from EZ (10) propagates to most brain regions after some delays (left). By eliminating target edges (right), seizure recruited regions are decreased significantly, even though the EZ generate seizure activities continuously.
(TIF)

S4 Fig Anatomical locations and simulation results for the optimal TZs (Patient FB7). **A** Anatomical locations of the TZs. Red nodes represent the EZ (72), yellow node (70) and green edge (70–79) indicate target node and target edge. **B** Network simulation results for safety verification. The results show the similarity coefficients between responsive activation patterns due to electrical stimulation before and after removing TZs. **C** Network simulation results for effectiveness verification. Before removing TZs, the seizure activity occurred from EZ (72) propagates to most brain regions after some delays (left). By eliminating the target node (middle) or the target edge (right), seizure recruited regions are decreased significantly, even though the EZ generate seizure activities continuously.
(TIF)

S5 Fig Anatomical locations and simulation results for the optimal TZs (Patient FB6). **A** Anatomical locations of the TZs. Red nodes represent EZs (48, 60, 81), yellow node (83) and green edges (83–45 83–72 83–78) indicate the target node and target edges. **B** Network simulation results for safety verification. The results show the similarity coefficients between responsive activation patterns due to electrical stimulation before and after removing TZs. **C** Network simulation results for effectiveness verification. Before removing TZs, the seizure activity occurred from EZs (48, 60, 81) propagates to most brain regions after some delays (left). By eliminating target nodes (middle) or target edges (right), seizure recruited regions are decreased significantly, even though the EZs generate seizure activities continuously.
(TIF)

S6 Fig Anatomical locations and simulation results for the optimal TZs (Patient PC). **A** Anatomical locations of the TZs. Red nodes represent EZs (47, 54, 55, 81), yellow nodes (63, 84) and green edges (57–63, 84–35) indicate target nodes and target edges. **B** Network simulation results for safety verification. The results show the similarity coefficients between responsive activation patterns due to electrical stimulation before and after removing TZs. **C** Network simulation results for effectiveness verification. Before removing TZs, the seizure activity occurred from EZs (47, 54, 55, 81) propagates to most brain regions after some delays (left). By eliminating target nodes (middle) or target edges (right), seizure recruited regions are decreased significantly, even though the EZs generate seizure activities continuously.
(TIF)

S7 Fig Anatomical locations and simulation results for the optimal TZ (Patient CV). **A** Anatomical locations of the TZs. Red nodes represent EZs (3, 22, 27), green edges (26–19, 76–44, 76–72, 76–52, 76–75, 23–21) indicate target edges. In this case, effective and safe target nodes for resection surgery are not obtained. **B** Network simulation results for safety verification. The results show the similarity coefficients between responsive activation patterns due to electrical stimulation before and after removing TZs. **C** Network simulation results for effectiveness verification. Before removing TZs, the seizure activity occurred from EZs (3, 22, 27) propagates to most brain regions after some delays (left). By eliminating target edges (right), seizure recruited regions are decreased significantly, even though the EZs generate seizure activities continuously.
(TIF)

S8 Fig Anatomical locations and simulation results for the optimal TZs (Patient SF). **A** Anatomical locations of the TZs. Red nodes represent EZs (53, 59, 61, 69), yellow nodes (6, 28, 30, 57, 73) and green edges (73–77, 84–64, 24–28, 57–63, 84–12, 84–35) indicate target nodes and target edges. **B** Network simulation results for safety verification. The results show the similarity coefficients between responsive activation patterns due to electrical stimulation before and after removing TZs. **C** Network simulation results for effectiveness verification. Before removing TZs, the seizure activity occurred from EZs (53, 59, 61, 69) propagates to most brain regions after some delays (left). By eliminating target nodes (middle) or target edges (right), seizure recruited regions are decreased significantly, even though the EZs generate seizure activities continuously.
(TIF)

S9 Fig TZs depending on the location of EZ (Patient IL). Cumulative results of **A** target nodes and **B** target edges that are derived according to the location of EZ. The result identifies several nodes and edges frequently used as TZ. Here, the resolution parameter for the modularity analysis is set to 1.0. **C** Anatomical locations of the nodes and the edges frequently obtained as TZ. The color code of nodes and the thickness of edges indicate the frequency used as TZ.
(TIF)

S10 Fig TZs depending on the location of EZ (Patient CJ). Cumulative results of **A** target nodes and **B** target edges that are derived according to the location of EZ. The result identifies several nodes and edges frequently used as TZ. Here, the resolution parameter for the modularity analysis is set to 1.0. **C** Anatomical locations of the nodes and the edges frequently obtained as TZ. The color code of nodes and the thickness of edges indicate the frequency used as TZ.
(TIF)

S11 Fig TZs depending on the location of EZ (Patient FB7). Cumulative results of **A** target nodes and **B** target edges that are derived according to the location of EZ. The result identifies several nodes and edges frequently used as TZ. Here, the resolution parameter for the modularity analysis is set to 1.0. **C** Anatomical locations of the nodes and the edges frequently obtained as TZ. The color code of nodes and the thickness of edges indicate the frequency used as TZ.
(TIF)

S12 Fig TZs depending on the location of EZ (Patient FB6). Cumulative results of **A** target nodes and **B** target edges that are derived according to the location of EZ. The result identifies several nodes and edges frequently used as TZ. Here, the resolution parameter for the modularity analysis is set to 1.0. **C** Anatomical locations of the nodes and the edges frequently obtained as TZ. The color code of nodes and the thickness of edges indicate the frequency used as TZ.
(TIF)

S13 Fig TZs depending on the location of EZ (Patient PC). Cumulative results of **A** target nodes and **B** target edges that are derived according to the location of EZ. The result identifies several nodes and edges frequently used as TZ. Here, the resolution parameter for the modularity analysis is set to 1.0. **C** Anatomical locations of the nodes and the edges frequently obtained as TZ. The color code of nodes and the thickness of edges indicate the frequency used as TZ.
(TIF)

S14 Fig TZs depending on the location of EZ (Patient SF). Cumulative results of **A** target nodes and **B** target edges that are derived according to the location of EZ. The result identifies several nodes and edges frequently used as TZ. Here, the resolution parameter for the modularity analysis is set to 1.0. **C** Anatomical locations of the nodes and the edges frequently obtained as TZ. The color code of nodes and the thickness of edges indicate the frequency used as TZ.
(TIF)
