# Supplementary figures and images for: Optimization of surgical intervention outside the epileptogenic zone in the Virtual Epileptic Patient (VEP)
Source: PLoS Comput Biol. 2019 Jun 26;15(6):e1007051. doi: 10.1371/journal.pcbi.1007051 (PMC6594587; doi:10.1371/journal.pcbi.1007051)

**S2 Table.**


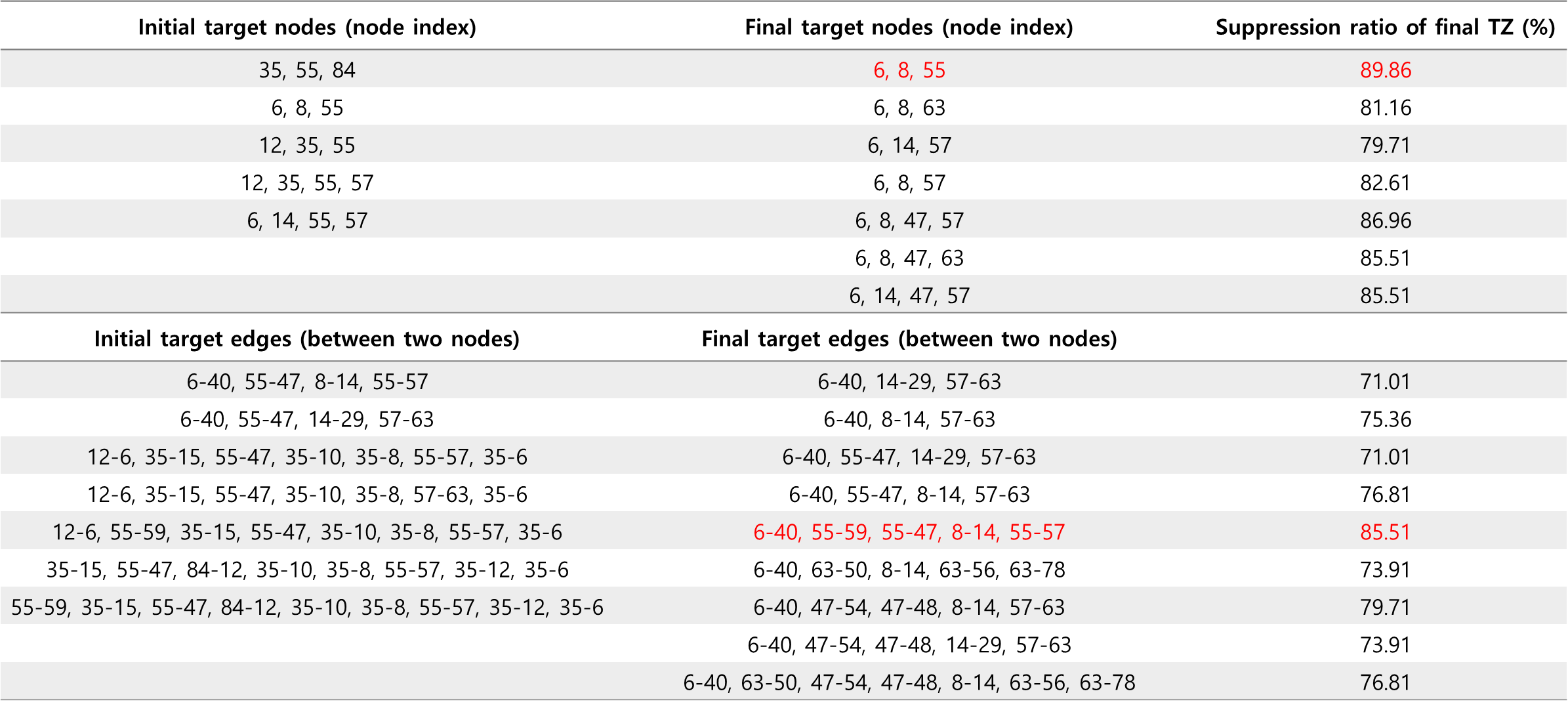

Supplement: S2 Table — (DOCX) [file pcbi.1007051.s002.docx]

**S3 Table.**


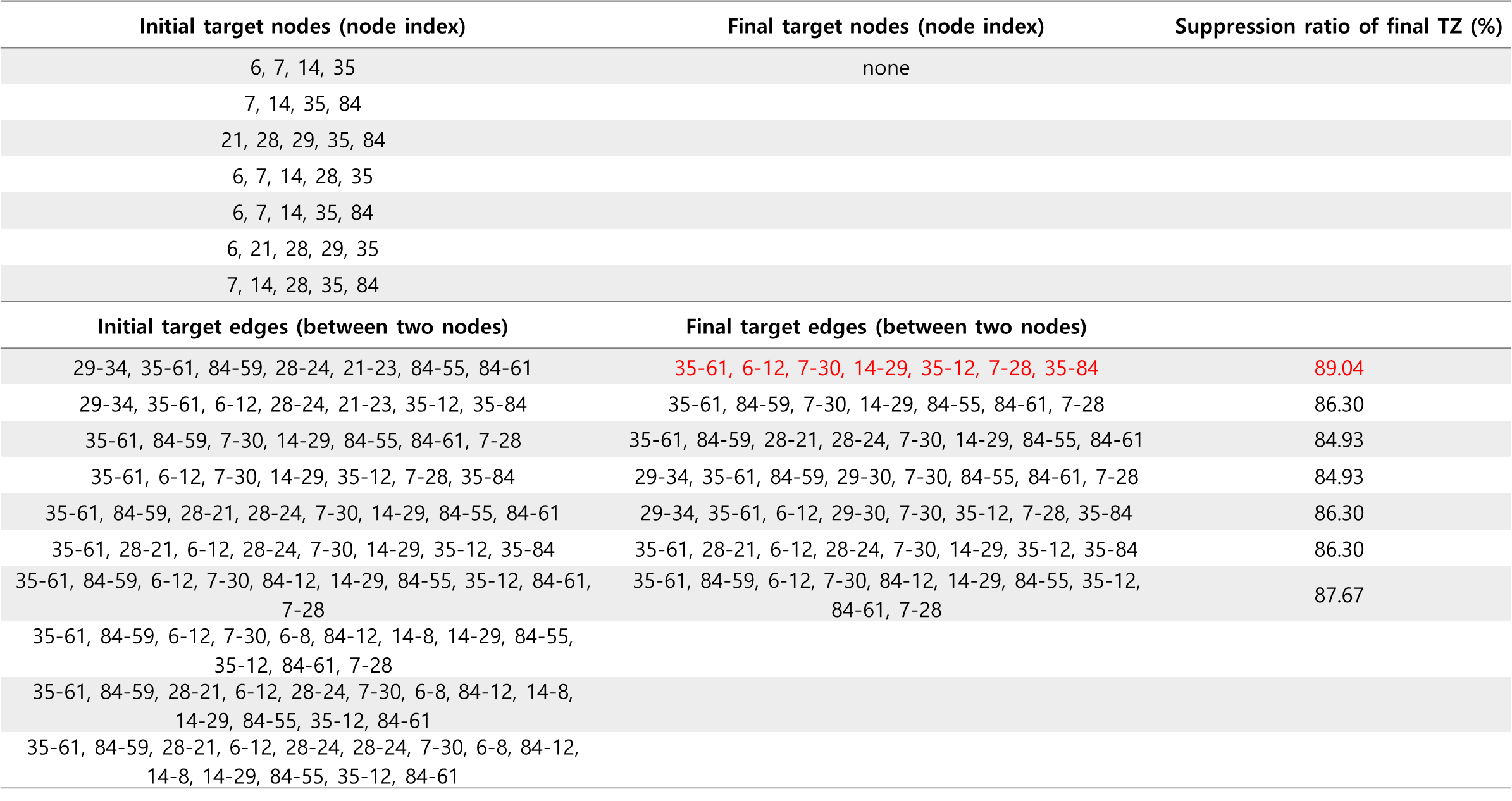

Supplement: S3 Table — (DOCX) [file pcbi.1007051.s003.docx]

**S4 Table.**


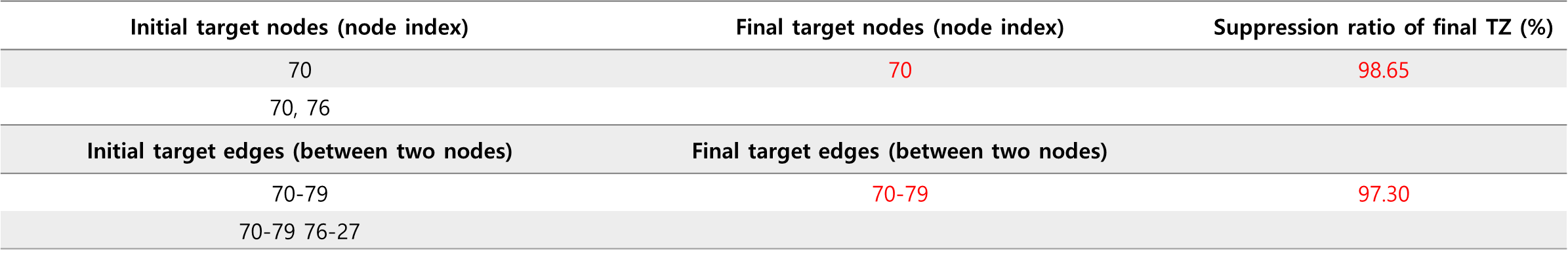

Supplement: S4 Table — (DOCX) [file pcbi.1007051.s004.docx]

**S5 Table.**


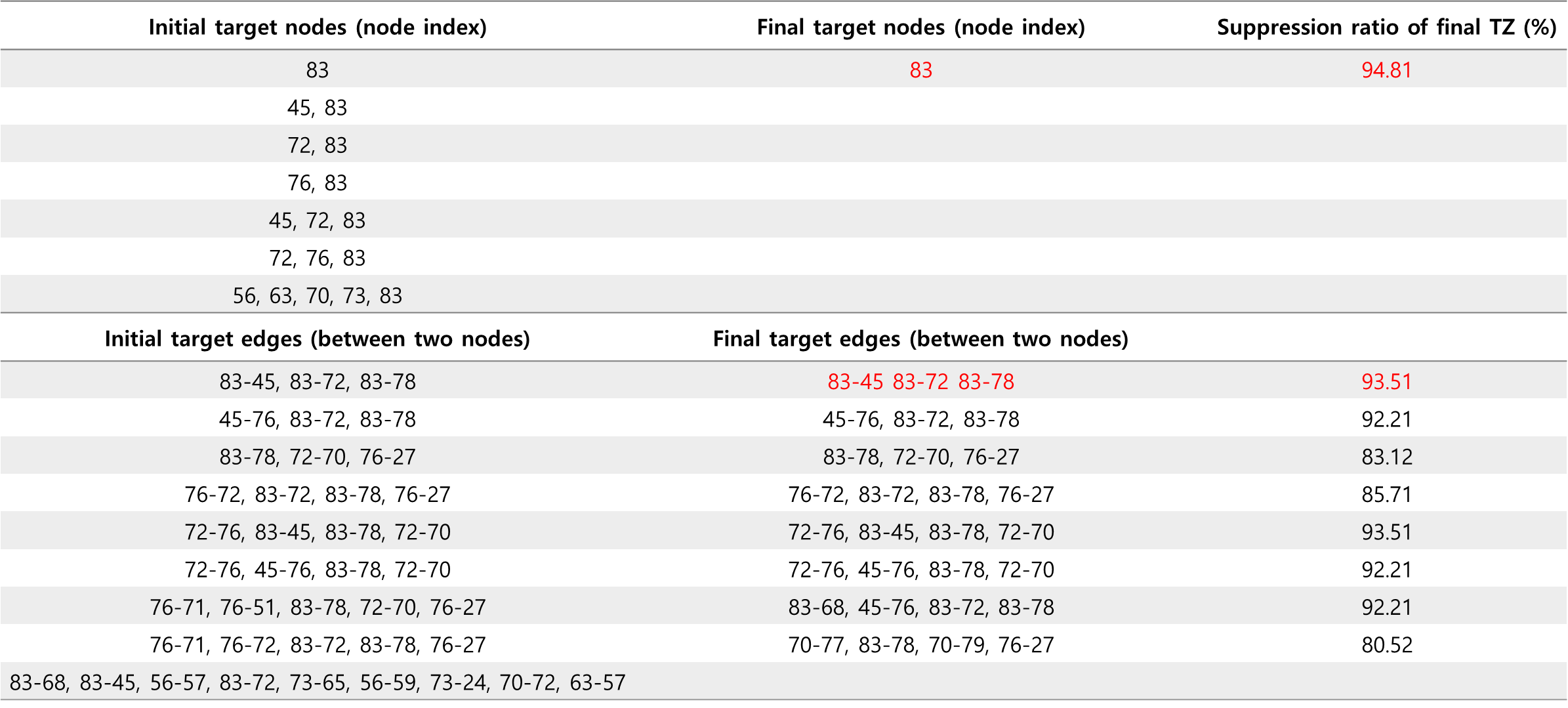

Supplement: S5 Table — (DOCX) [file pcbi.1007051.s005.docx]

**S6 Table.**


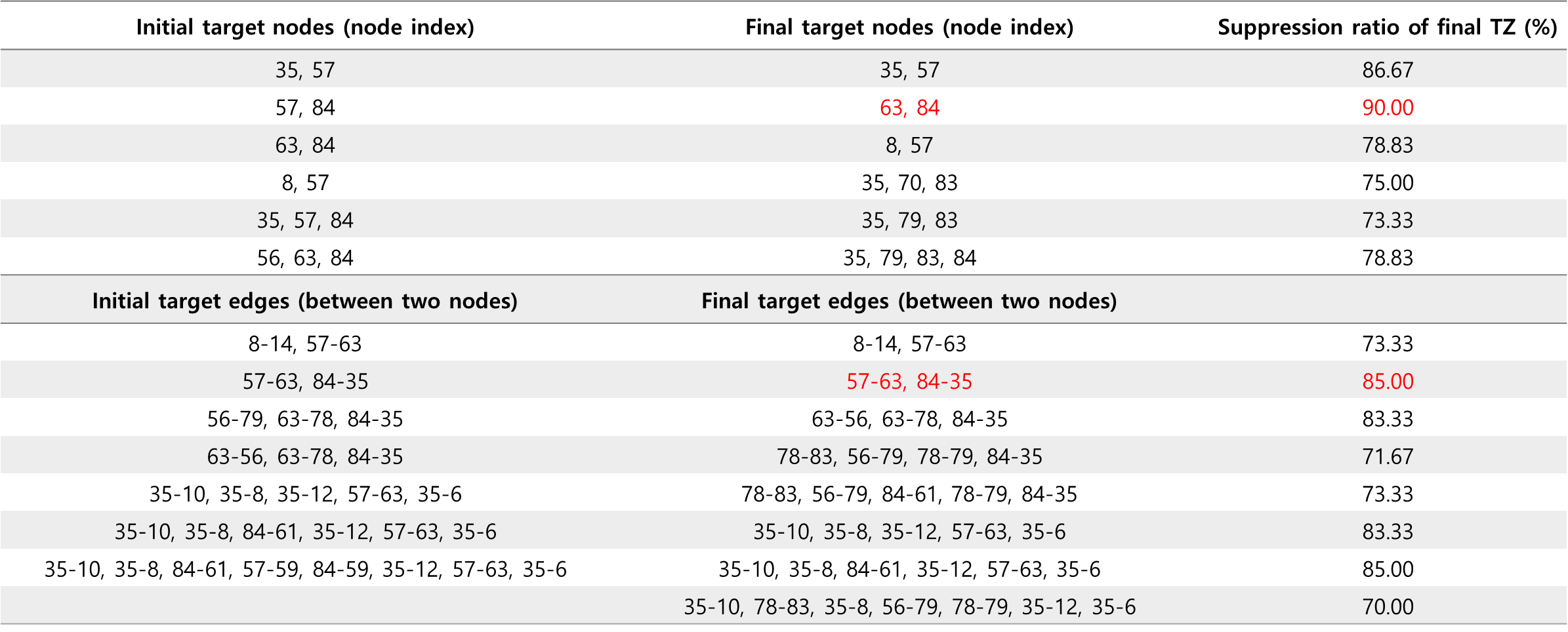

Supplement: S6 Table — (DOCX) [file pcbi.1007051.s006.docx]

**S7 Table.**


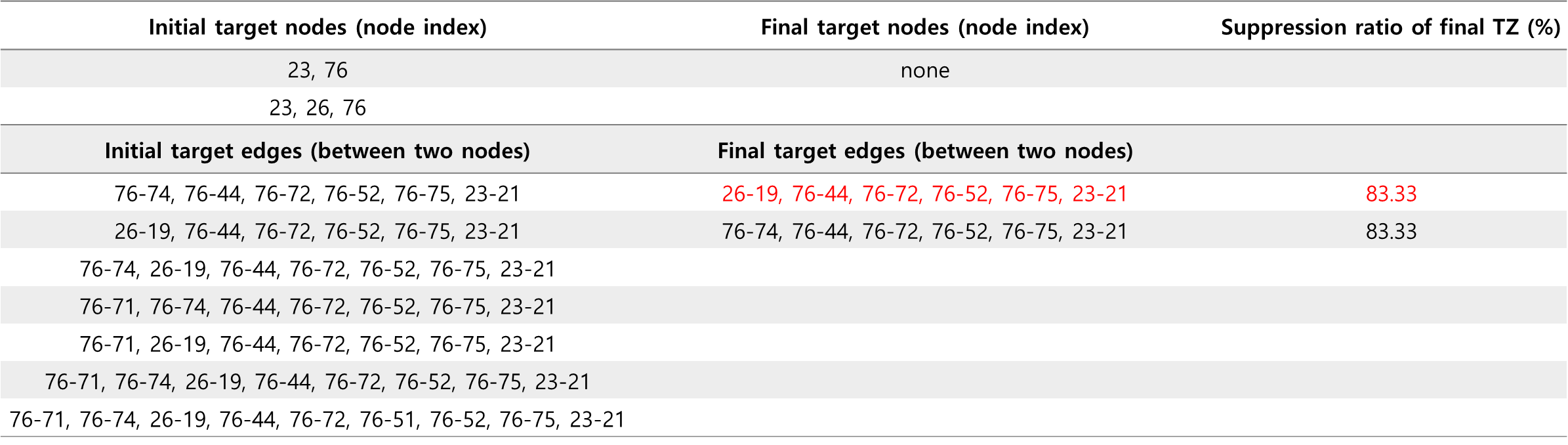

Supplement: S7 Table — (DOCX) [file pcbi.1007051.s007.docx]

**S8 Table.**


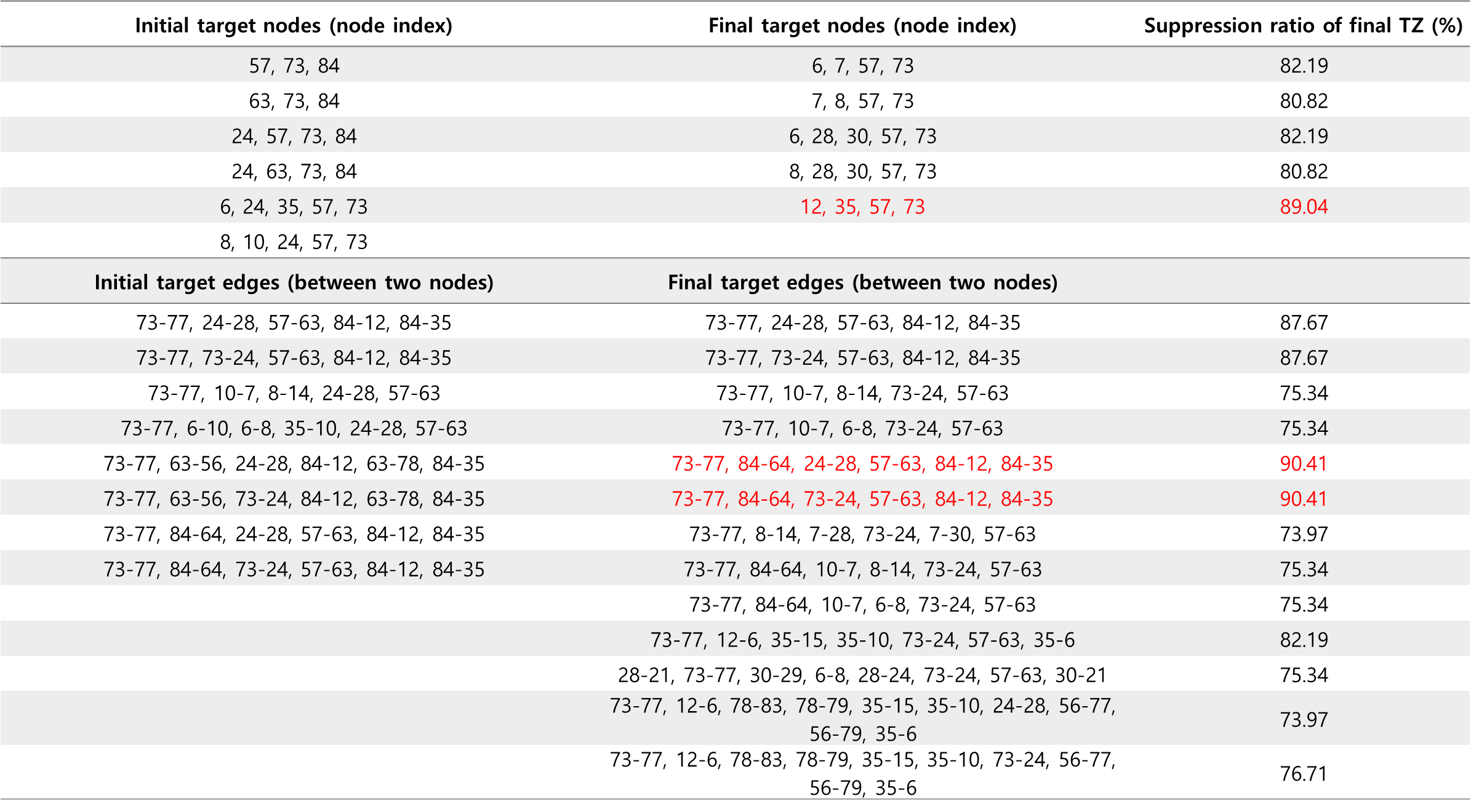

Supplement: S8 Table — (DOCX) [file pcbi.1007051.s008.docx]

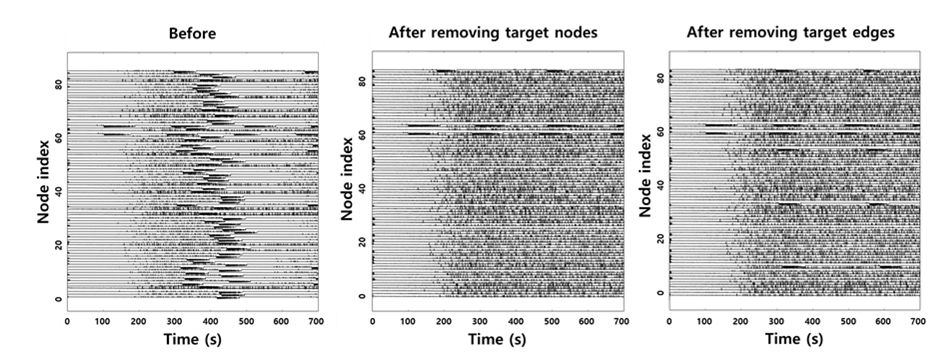

Supplement: S1 Fig — The figures show propagation characteristics of the seizure occurred from EZs (node 61 and 64) before (left) and after eliminating target nodes (middle) or target edges (right). (TIF) [file pcbi.1007051.s010.tif]

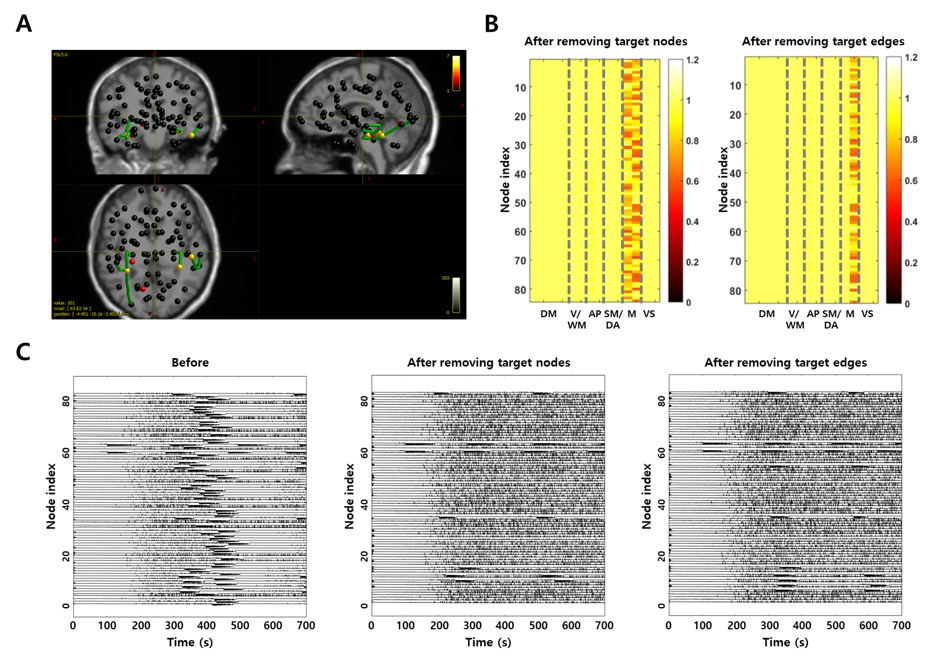

Supplement: S2 Fig — A Anatomical locations of the TZs. Red nodes represent EZs (61, 64), yellow node (6, 8, 55) and green edge (6–40, 55–59, 55–47, 8–14, 55–57) indicate target node and target edge. B Network simulation results for safety verification. The results show the similarity coefficients between responsive activation patterns due to electrical stimulation before and after removing TZs. C Network simulation results for effectiveness verification. Before removing TZs, the seizure activity occurred from EZs (61, 64) propagates to most brain regions after some delays (left). By eliminating the target node (middle) or the target edge (right), seizure recruited regions are decreased significantly, even though the EZ generate seizure activities continuously. (TIF) [file pcbi.1007051.s011.tif]

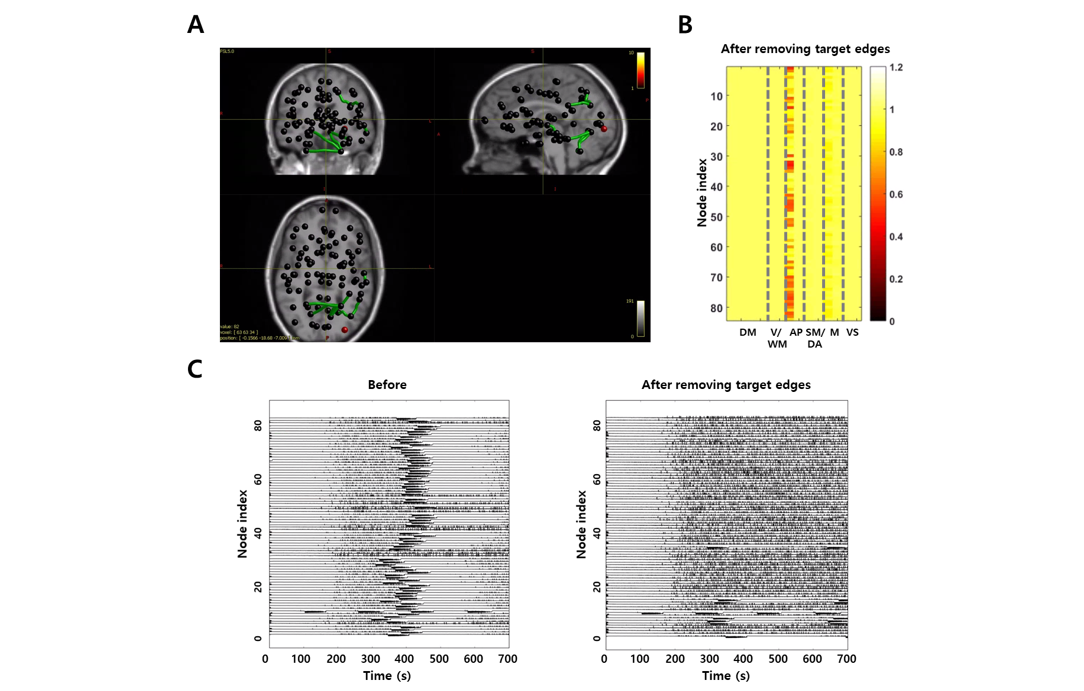

Supplement: S3 Fig — A Anatomical locations of the TZ. Red nodes represent the EZ (10), green edges (35–61, 6–12, 7–30, 14–29, 35–12, 7–28, 35–84) indicate target edges. In this case, effective and safe target nodes for resection surgery are not obtained. B Network simulation results for safety verification. The results show the similarity coefficients between responsive activation patterns due to electrical stimulation before and after removing TZs. C Network simulation results for effectiveness verification. Before removing TZs, the seizure activity occurred from EZ (10) propagates to most brain regions after some delays (left). By eliminating target edges (right), seizure recruited regions are decreased significantly, even though the EZ generate seizure activities continuously. (TIF) [file pcbi.1007051.s012.tif]

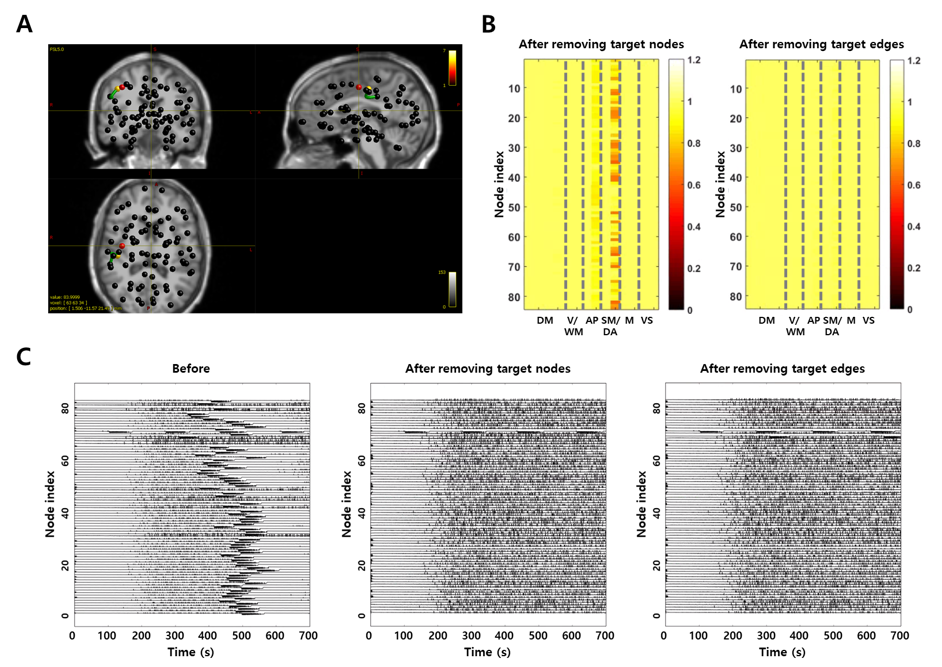

Supplement: S4 Fig — A Anatomical locations of the TZs. Red nodes represent the EZ (72), yellow node (70) and green edge (70–79) indicate target node and target edge. B Network simulation results for safety verification. The results show the similarity coefficients between responsive activation patterns due to electrical stimulation before and after removing TZs. C Network simulation results for effectiveness verification. Before removing TZs, the seizure activity occurred from EZ (72) propagates to most brain regions after some delays (left). By eliminating the target node (middle) or the target edge (right), seizure recruited regions are decreased significantly, even though the EZ generate seizure activities continuously. (TIF) [file pcbi.1007051.s013.tif]

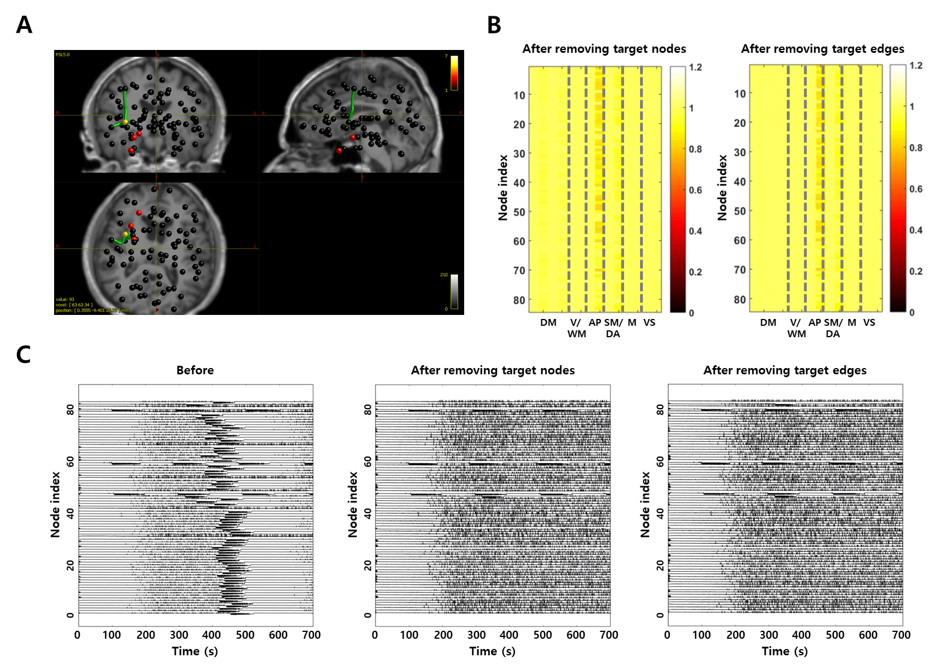

Supplement: S5 Fig — A Anatomical locations of the TZs. Red nodes represent EZs (48, 60, 81), yellow node (83) and green edges (83–45 83–72 83–78) indicate the target node and target edges. B Network simulation results for safety verification. The results show the similarity coefficients between responsive activation patterns due to electrical stimulation before and after removing TZs. C Network simulation results for effectiveness verification. Before removing TZs, the seizure activity occurred from EZs (48, 60, 81) propagates to most brain regions after some delays (left). By eliminating target nodes (middle) or target edges (right), seizure recruited regions are decreased significantly, even though the EZs generate seizure activities continuously. (TIF) [file pcbi.1007051.s014.tif]

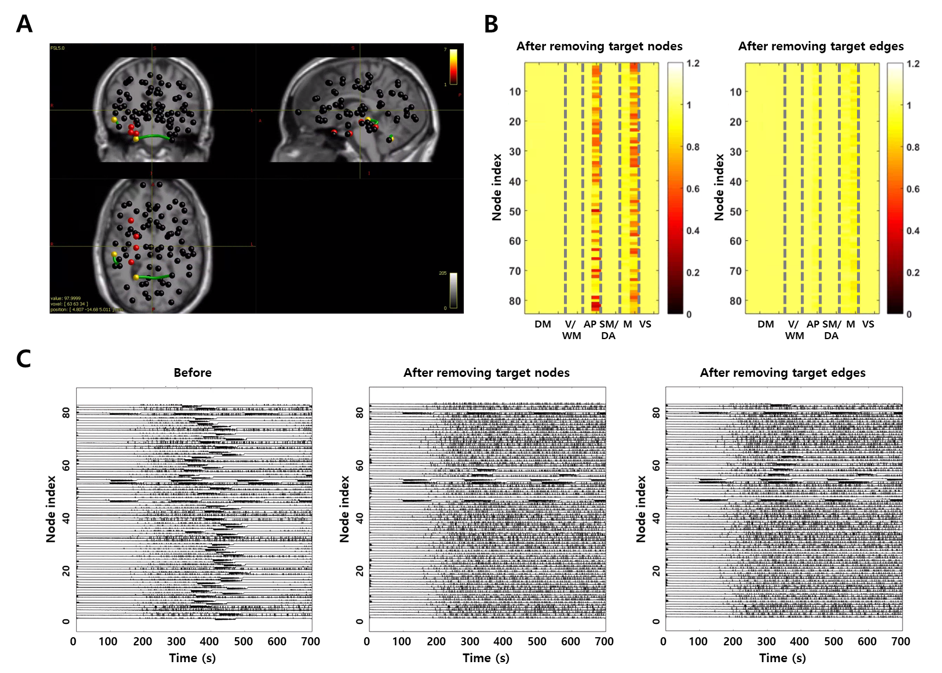

Supplement: S6 Fig — A Anatomical locations of the TZs. Red nodes represent EZs (47, 54, 55, 81), yellow nodes (63, 84) and green edges (57–63, 84–35) indicate target nodes and target edges. B Network simulation results for safety verification. The results show the similarity coefficients between responsive activation patterns due to electrical stimulation before and after removing TZs. C Network simulation results for effectiveness verification. Before removing TZs, the seizure activity occurred from EZs (47, 54, 55, 81) propagates to most brain regions after some delays (left). By eliminating target nodes (middle) or target edges (right), seizure recruited regions are decreased significantly, even though the EZs generate seizure activities continuously. (TIF) [file pcbi.1007051.s015.tif]

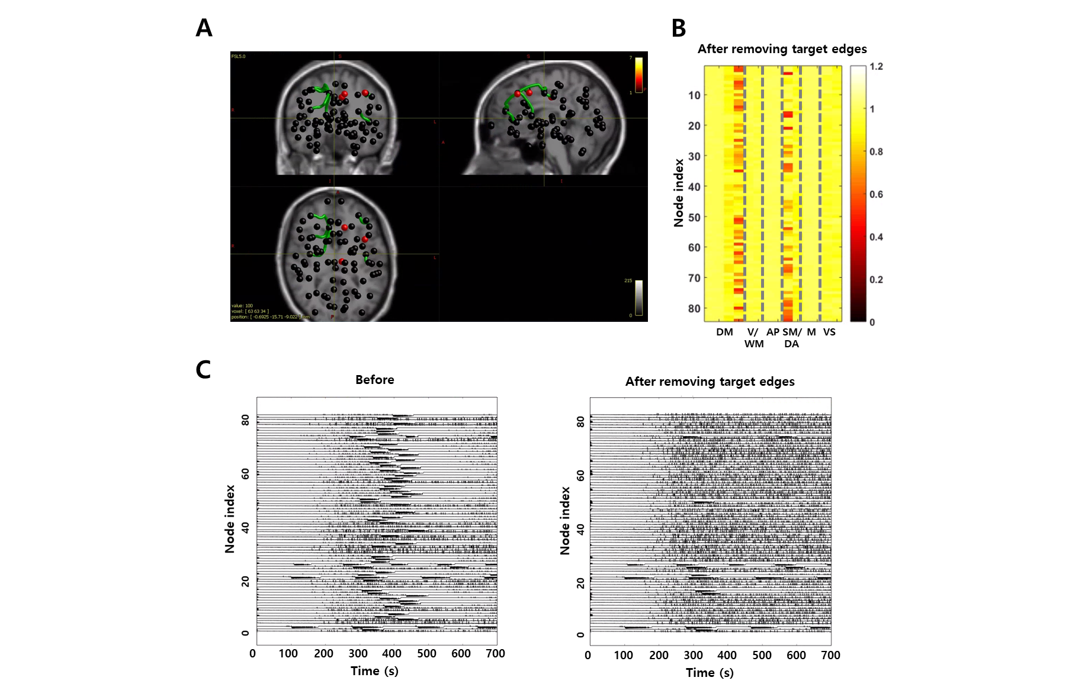

Supplement: S7 Fig — A Anatomical locations of the TZs. Red nodes represent EZs (3, 22, 27), green edges (26–19, 76–44, 76–72, 76–52, 76–75, 23–21) indicate target edges. In this case, effective and safe target nodes for resection surgery are not obtained. B Network simulation results for safety verification. The results show the similarity coefficients between responsive activation patterns due to electrical stimulation before and after removing TZs. C Network simulation results for effectiveness verification. Before removing TZs, the seizure activity occurred from EZs (3, 22, 27) propagates to most brain regions after some delays (left). By eliminating target edges (right), seizure recruited regions are decreased significantly, even though the EZs generate seizure activities continuously. (TIF) [file pcbi.1007051.s016.tif]

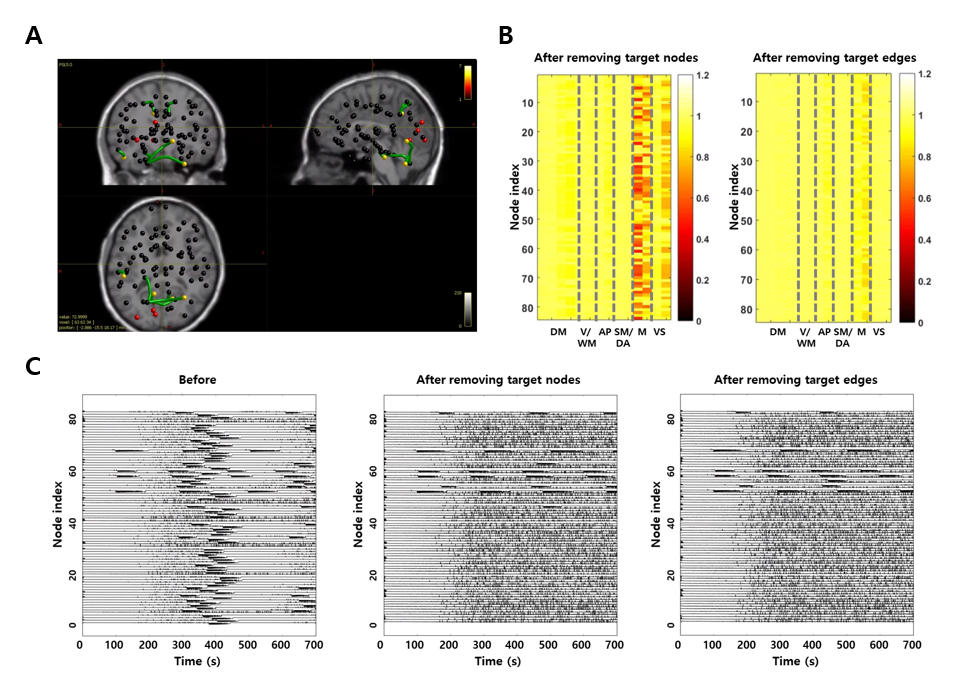

Supplement: S8 Fig — A Anatomical locations of the TZs. Red nodes represent EZs (53, 59, 61, 69), yellow nodes (6, 28, 30, 57, 73) and green edges (73–77, 84–64, 24–28, 57–63, 84–12, 84–35) indicate target nodes and target edges. B Network simulation results for safety verification. The results show the similarity coefficients between responsive activation patterns due to electrical stimulation before and after removing TZs. C Network simulation results for effectiveness verification. Before removing TZs, the seizure activity occurred from EZs (53, 59, 61, 69) propagates to most brain regions after some delays (left). By eliminating target nodes (middle) or target edges (right), seizure recruited regions are decreased significantly, even though the EZs generate seizure activities continuously. (TIF) [file pcbi.1007051.s017.tif]

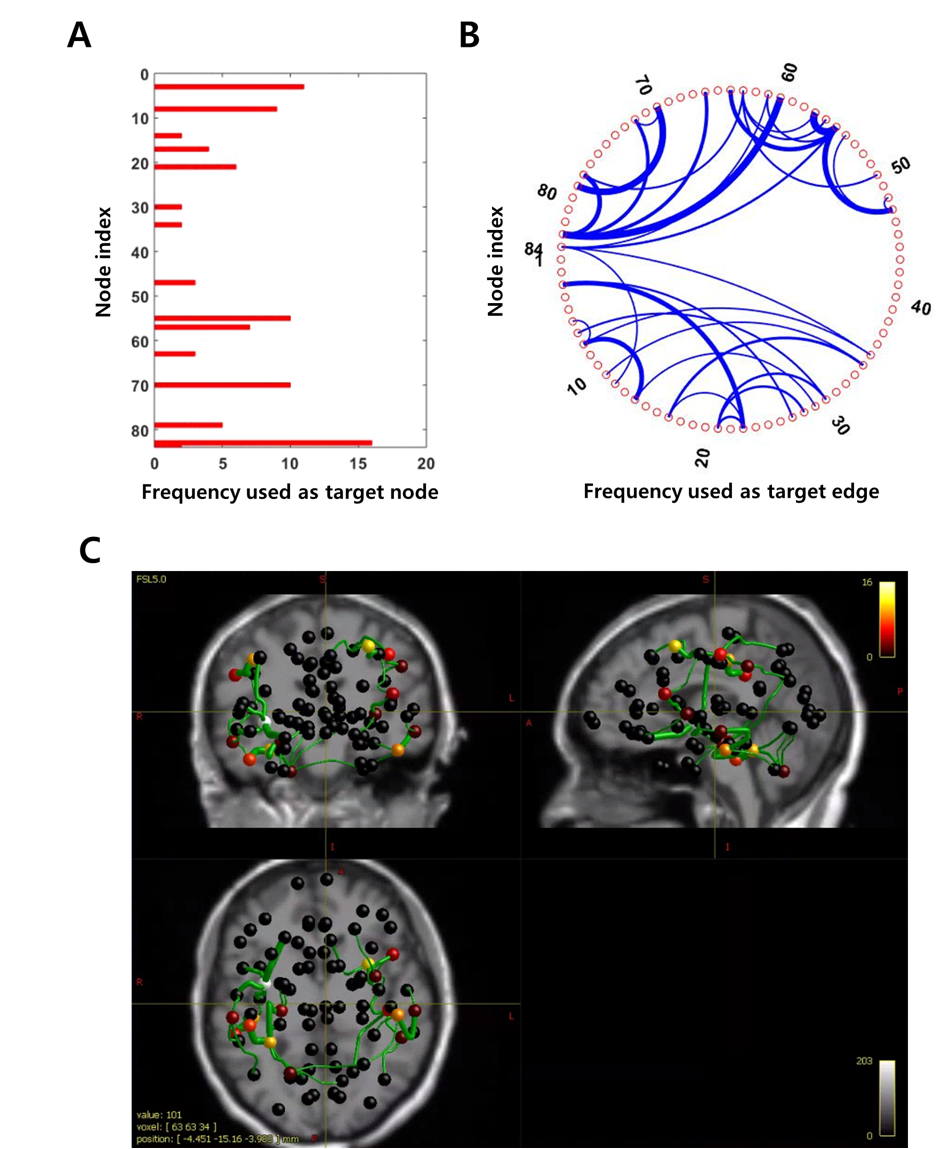

Supplement: S9 Fig — Cumulative results of A target nodes and B target edges that are derived according to the location of EZ. The result identifies several nodes and edges frequently used as TZ. Here, the resolution parameter for the modularity analysis is set to 1.0. C Anatomical locations of the nodes and the edges frequently obtained as TZ. The color code of nodes and the thickness of edges indicate the frequency used as TZ. (TIF) [file pcbi.1007051.s018.tif]

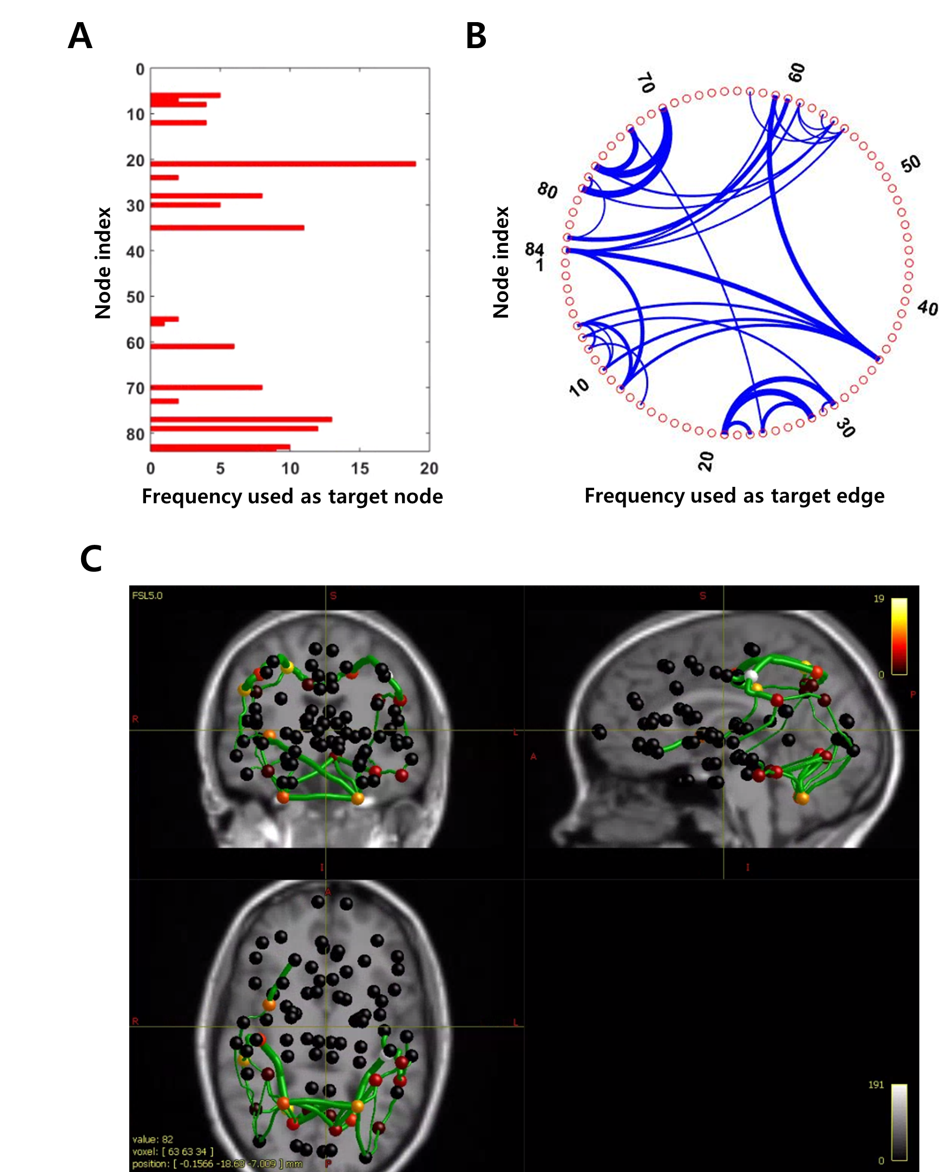

Supplement: S10 Fig — Cumulative results of A target nodes and B target edges that are derived according to the location of EZ. The result identifies several nodes and edges frequently used as TZ. Here, the resolution parameter for the modularity analysis is set to 1.0. C Anatomical locations of the nodes and the edges frequently obtained as TZ. The color code of nodes and the thickness of edges indicate the frequency used as TZ. (TIF) [file pcbi.1007051.s019.tif]

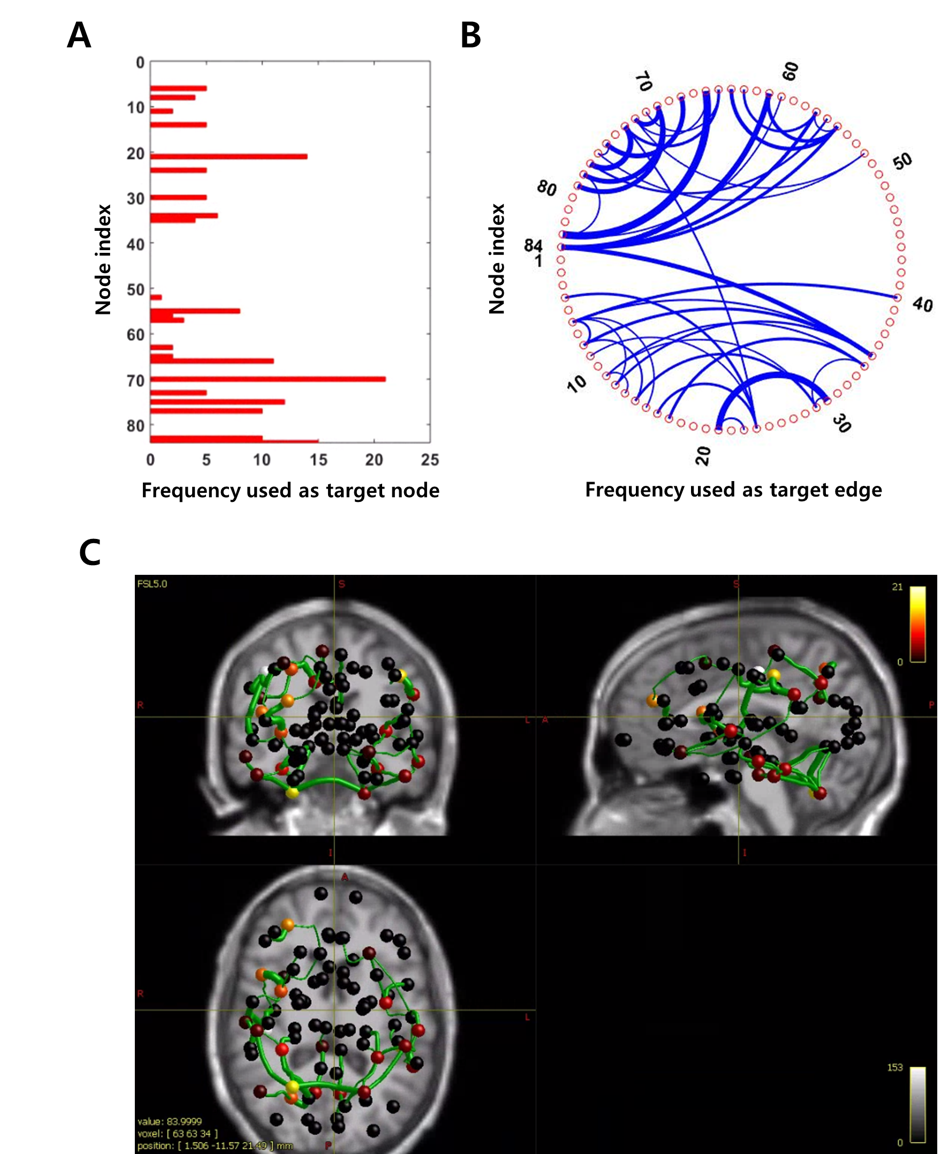

Supplement: S11 Fig — Cumulative results of A target nodes and B target edges that are derived according to the location of EZ. The result identifies several nodes and edges frequently used as TZ. Here, the resolution parameter for the modularity analysis is set to 1.0. C Anatomical locations of the nodes and the edges frequently obtained as TZ. The color code of nodes and the thickness of edges indicate the frequency used as TZ. (TIF) [file pcbi.1007051.s020.tif]

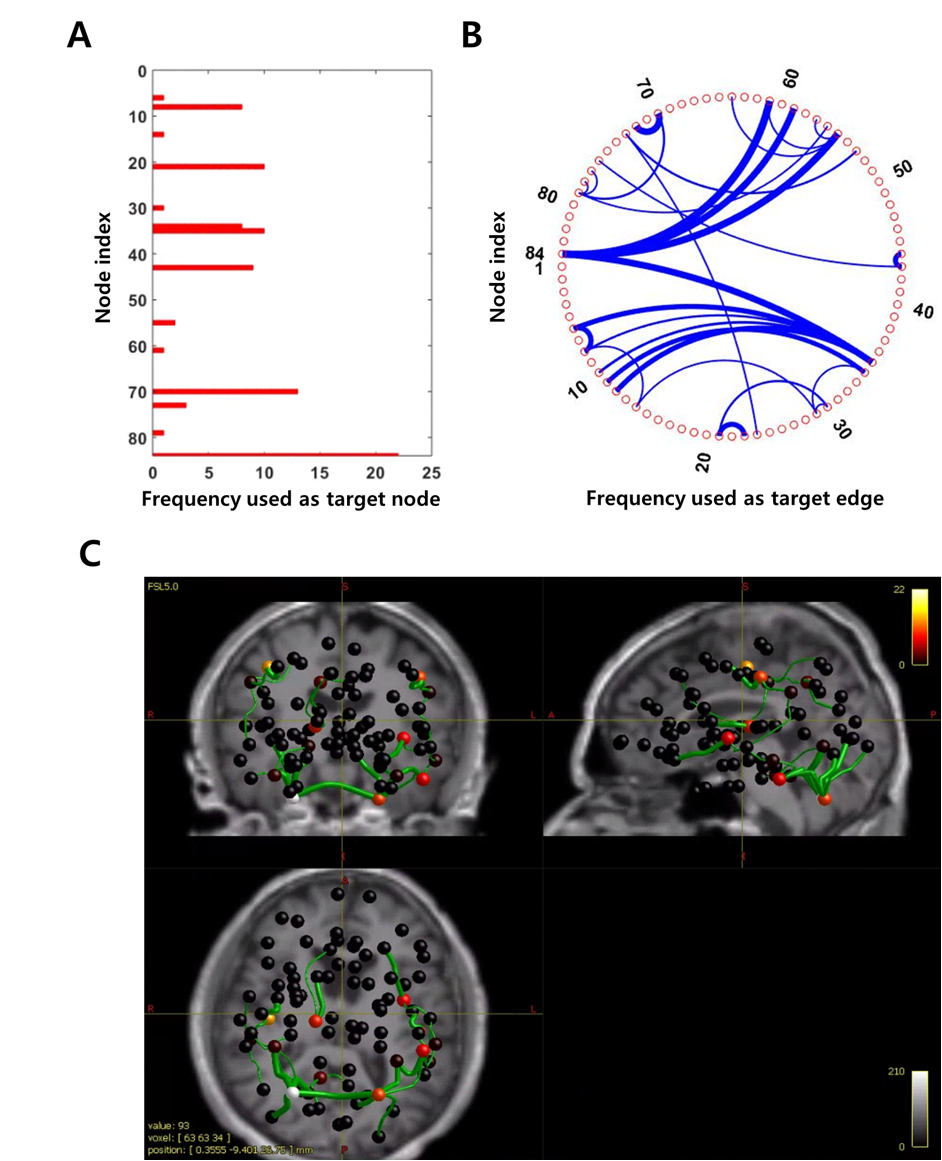

Supplement: S12 Fig — Cumulative results of A target nodes and B target edges that are derived according to the location of EZ. The result identifies several nodes and edges frequently used as TZ. Here, the resolution parameter for the modularity analysis is set to 1.0. C Anatomical locations of the nodes and the edges frequently obtained as TZ. The color code of nodes and the thickness of edges indicate the frequency used as TZ. (TIF) [file pcbi.1007051.s021.tif]

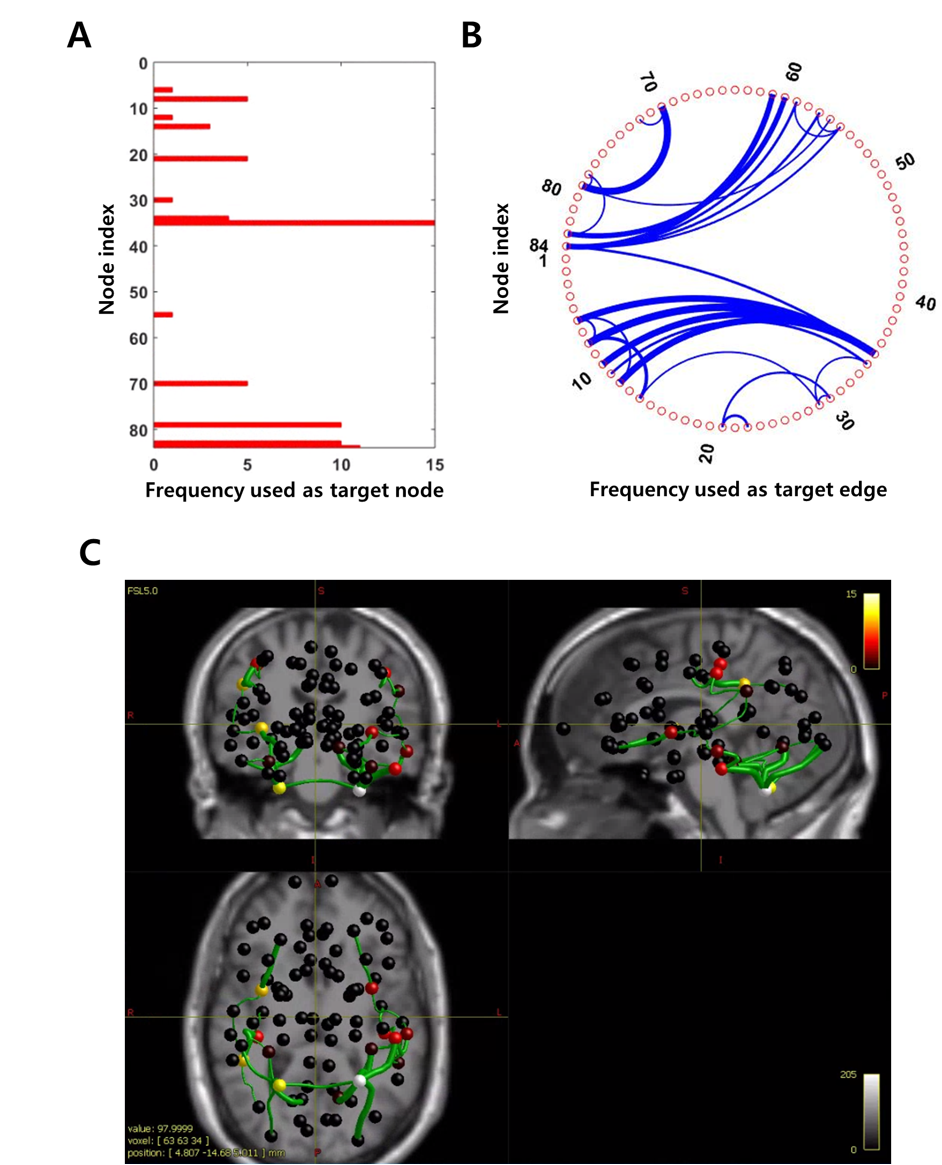

Supplement: S13 Fig — Cumulative results of A target nodes and B target edges that are derived according to the location of EZ. The result identifies several nodes and edges frequently used as TZ. Here, the resolution parameter for the modularity analysis is set to 1.0. C Anatomical locations of the nodes and the edges frequently obtained as TZ. The color code of nodes and the thickness of edges indicate the frequency used as TZ. (TIF) [file pcbi.1007051.s022.tif]

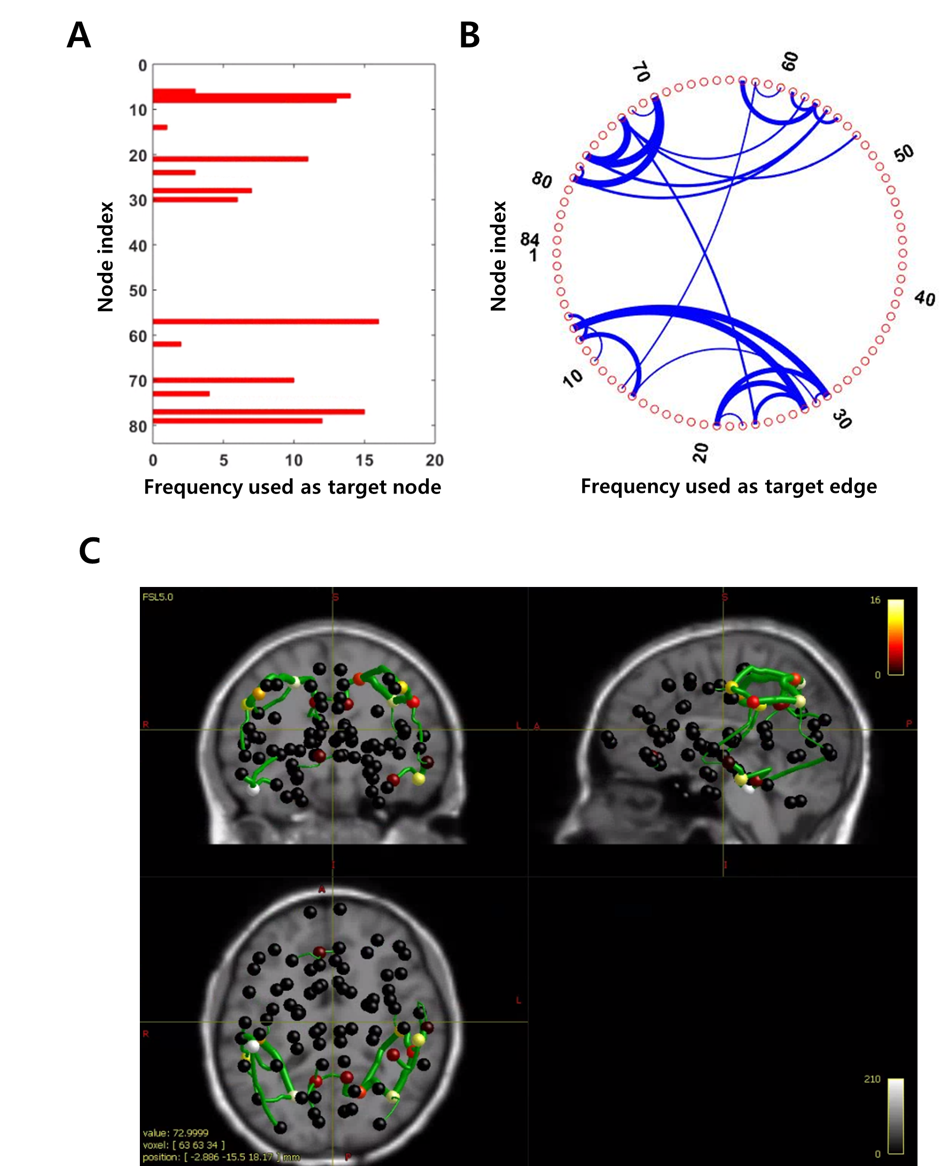

Supplement: S14 Fig — Cumulative results of A target nodes and B target edges that are derived according to the location of EZ. The result identifies several nodes and edges frequently used as TZ. Here, the resolution parameter for the modularity analysis is set to 1.0. C Anatomical locations of the nodes and the edges frequently obtained as TZ. The color code of nodes and the thickness of edges indicate the frequency used as TZ. (TIF) [file pcbi.1007051.s023.tif]
