# Supplementary material for: Optimization of surgical intervention outside the epileptogenic zone in the Virtual Epileptic Patient (VEP)
Source: PLoS Comput Biol. 2019 Jun 26;15(6):e1007051. doi: 10.1371/journal.pcbi.1007051 (PMC6594587; doi:10.1371/journal.pcbi.1007051)
Supplement: S9 Table — Th, thermocoagulation; Gk, Gamma knife; Sr, surgical resection; NO, not operated; N, normal; FCD, focal cortical dysplasia; SPC, superior parietal cortex; Fr, Frontal; PVH, periventricular nodular heterotopia; NA, not available; L, left; R, right (From [37]). (DOCX) [file pcbi.1007051.s009.docx]

**S9 Table.**
